# Highly Biomimetic Ectodermal Epithelial Organoids for Epithelial Barrier Stimulation Assays

**DOI:** 10.1002/advs.202522709

**Published:** 2026-03-28

**Authors:** Yiming Chen, Yuman Li, Chenyu Deng, Leran Li, Zhewen Hu, Boon Chin Heng, Yuting Niu, Shiyu Sun, Min Gao, Xiaopei Chi, Ying He, Mingming Xu, Ying Huang, Xuliang Deng

**Affiliations:** ^1^ Department of Geriatric Dentistry Peking University School and Hospital of Stomatology & National Center of Stomatology & National Clinical Research Center for Oral Diseases & National Engineering Research Center of Oral Biomaterials and Digital Medical Devices & Beijing Key Laboratory of Biomaterials for Oral Disease Beijing P. R. China; ^2^ Department of Orthodontics Peking University School and Hospital of Stomatology & National Center for Stomatology & National Clinical Research Center for Oral Diseases & National Engineering Research Center of Oral Biomaterials and Digital Medical Devices Beijing P. R. China; ^3^ Central Laboratory Peking University School and Hospital of Stomatology Beijing P. R. China; ^4^ School of Medical and Life Sciences Sunway University Darul Ehsan Selangor Malaysia

**Keywords:** ectodermal epithelial organoids (EEOs), epithelial barrier stimulation tests, intercellular junctions, stratum corneum, TGF‐β signaling pathway

## Abstract

Evaluating the potential toxicity of pharmaceuticals and biomaterials to ectodermal epithelia, such as the oral mucosa and skin, is indispensable in pre‐clinical assessments. However, this remains a challenge primarily owing to the lack of physiologically relevant and accurate screening models. To address this need, we referred to developmental patterns of the ectodermal epithelium in vivo and adopted the strategic downregulation of the TGF‐β signaling pathway in organoid fabrication. Our novel ectodermal epithelial organoids (EEOs) recapitulate the cellular and histological complexities of native epithelial tissues and improve fabrication efficiency. EEOs can accurately capture essential barrier components, including the stratum corneum and intercellular junctions, which are crucial for maintaining epithelial barrier integrity. Validation studies revealed that EEOs are capable of modeling the dose‐dependent cytotoxic effects of pharmaceuticals on the oral mucosa and identifying previously undetectable nanomaterial‐induced damage to intercellular junctions. These findings indicate that the model system is a robust platform for epithelial barrier stimulation tests, and that this technology is poised to transform pre‐clinical assessment paradigms by enabling more accurate toxicity prediction and accelerating the development of safer pharmaceuticals and biomaterials.

## 1 Introduction

The ectodermal epithelium, consisting of the skin, oral mucosa, and corneal epithelia, separates the underlying tissues from the external environment [1]. In the application of topical agents, dental medications, and cosmetics, the ectodermal epithelium acts as a primary barrier, in which the stratum corneum and cellular junctions play essential roles [1, 2, 3, 4]. Thus, stimulation assays of the ectodermal epithelium are key procedures in pre‐clinical screening of innovative pharmaceuticals and biomaterials [5]. Currently, in vitro stimulation assays, predominantly based on 2D cultured keratinocytes, are incapable of recapitulating the cell subtypes, histological structures, and barrier functions of ectodermal epithelial tissues [6, 7]. Meanwhile, in vivo assays using experimental animals have inevitable shortcomings due to interspecies divergence and the inaccessibility of real‐time observation [8, 9]. Notably, the emerging technique of organoids illuminates an anticipated path for establishing a novel platform for pre‐clinical assessments of the ectodermal epithelial barrier [10, 11, 12, 13, 14, 15, 16].

Currently, studies on epithelial organoids predominantly focus on gastrointestinal tissues derived from the endoderm [17, 18, 19]. Due to divergent developmental patterns between germ layers, culture conditions that are well adapted for endodermal epithelial organoids are not suitable for the development of ectodermal epithelial organoids (EEOs) [20, 21]. Specifically, adopting previously reported methods (PRM) for establishing oral mucosal or skin organoids may lead to discrepancies in the regulation of critical signaling pathways, which in turn restrict the ability of organoids to recapitulate the features of native ectodermal epithelial tissues. In terms of histological characteristics, endodermal epithelial organoids are typically monolayered, consisting of fewer epithelial cell subtypes. In contrast, the ectodermal epithelium is characterized by a stratified structure that consists of a hierarchal arrangement of different epithelial subtypes, formation of a stratum corneum, and the establishment of intercellular junctions, such as E‐cadherin and desmoplakin [22, 23]. As a result, in previously reported organoids (PROs) derived from oral mucosal epithelial stem cells, the culture microenvironment, which is largely identical to that used for endodermal epithelial organoids (e.g., R‐spondin, noggin, EGF, b‐FGF, and FGF10), disadvantages the committed differentiation of stem cells and thus hampers the formation of crucial structures of the ectodermal epithelial barrier [17, 18]. Therefore, there is an urgent need for the establishment of EEOs capable of recapitulating the barrier functions of the ectodermal epithelium, which are essential for carrying out pre‐clinical assessments of pharmaceutics and biomaterials [12, 24, 25, 26].

Here, we propose a novel strategy for fabricating EEOs that closely recapitulate the natural developmental patterns of ectodermal epithelia. By examining molecular characteristics during the developmental process, we observed that the TGF‐β signaling pathway is significantly downregulated in the ectodermal epithelium, compared with the endodermal epithelium. By comparing the molecular features of native ectodermal tissues and PROs, we confirmed that markers related to the TGF‐β signaling pathway show the greatest divergence. Therefore, through the strategic downregulation of the TGF‐β signaling pathway, we fabricated organoids that faithfully represent the cell types and structural characteristics of the ectodermal epithelium, especially the stratum corneum and cellular junctions. The superiority of our strategy was demonstrated through two key applications. First, our EEOs sensitively detected subtle damage caused by nanomaterials to the ectodermal epithelial barrier junctions, an effect that has been overlooked by previous assessment methods. Second, when evaluating the response of ectodermal epithelia to chemical stimulation (e.g., Listerine), our EEOs accurately recapitulated the alterations in viability and gene expression patterns. To the best of our knowledge, this work is the first to develop an EEOs culture system that can be adopted for the comprehensive assessment of ectodermal epithelial barrier stimulation. The high physiological relevance and sensitivity of this system make it particularly valuable for evaluating both overt and subtle epithelial responses to barrier stimulation.

## 2 Results

### 2.1 Deciphering the Pivotal Role of TGF‐β Signaling Pathway Downregulation in the Fabrication of EEOs

**FIGURE 1:**
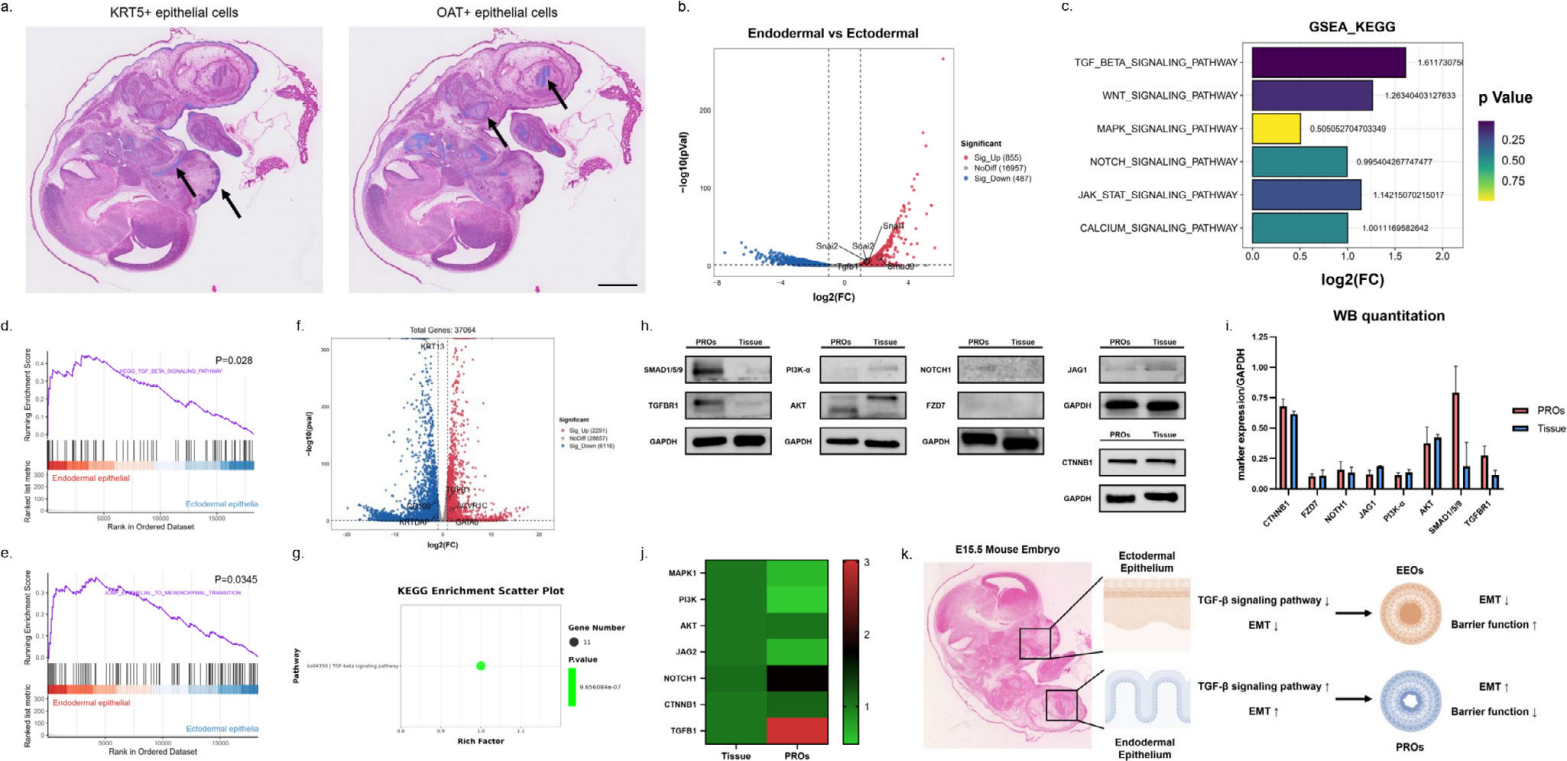
The role of downregulation of the TGF‐β signaling pathway in EEOs fabrication by modeling development a) Visium HD spatial transcriptomic analysis depicting KRT5+ and OAT+ epithelial cells (bar = 1000 µm). b) Volcano plot depicting DEGs between endodermal and ectodermal epithelial cells (critical marker genes are labeled in the plot). c) GSEA–KEGG analysis of DEGs between endodermal and ectodermal epithelial cells. d) GSEA–KEGG analysis of DEGs between endodermal and ectodermal epithelial cells showing significant upregulation of TGF‐β signaling pathway. e) GSEA–GO analysis of DEGs between endodermal and ectodermal epithelial cells showing significant upregulation of EMT. f) Volcano plot depicting DEGs between oral epithelial tissues and PROs (critical marker genes are labeled in the plot). g) KEGG analysis of significantly upregulated genes in PROs showing significant enrichment in TGF‐β signaling pathway. h) Representative western blotting images comparing relative expression levels of critical markers related to the Wnt, Notch, PI3K‐Akt, and TGF‐β signaling pathways. i) Western blotting quantitative analysis of expression levels of critical markers related to the Wnt, Notch, PI3K‐Akt, and TGF‐β signaling pathways (data are shown as means ± SEM; t‐test; n = 3). j) qRT‐PCR analysis of relative expression levels of critical genes related to the Wnt, Notch, PI3K‐Akt, and TGF‐β signaling pathways (data are shown as means ± SEM; t‐test; n = 3). k) Schematic diagram depicting variations in the regulation of the TGF‐β signaling pathway between ectodermal and endodermal epithelium.

To uncover pivotal cues for establishing an ectodermal epithelium–specific in vitro culture microenvironment, we examined the divergence between endodermal (e.g., intestine) and ectodermal (e.g., oral mucosa) epithelia during embryonic development. A KRT5^+^ oral mucosal epithelial cell subcluster and an OAT^+^ intestinal epithelial cell subcluster in an E15.5 mouse embryo were identified using Visium HD spatial transcriptomics (Figure 1a; Figure S1a,b). Differential enrichment analysis between the two subclusters showed that genes related to the TGF‐β signaling pathway, including *SNAI1*, *SNAI2*, *TGFB1*, and *SMAD9*, were significantly upregulated in intestinal epithelial cells, as compared with oral mucosal epithelial cells (Figure 1b; Figure S1c). Gene set enrichment analysis (GSEA) revealed that among crucial signaling pathways related to tissue development, the TGF‐β signaling pathway showed the greatest variation between ectodermal and endodermal epithelia (Figure 1c). Specifically, the TGF‐β signaling pathway was significantly upregulated in the endodermal epithelium (Figure 1d). Moreover, epithelial‐to‐mesenchymal transition (EMT) was also significantly enhanced in endodermal epithelia compared with ectodermal epithelia (Figure 1e; Figure S1d).

Furthermore, the divergence between PROs and native oral epithelial tissues was examined. RNA‐sequencing assays revealed significant variation in the regulation of the TGF‐β signaling pathway and the expression levels of critical markers related to EMT (Figure 1f,g). Western blotting and qRT‐PCR assays further confirmed that the TGF‐β signaling pathway showed the greatest variation between PROs and native oral epithelial tissues compared with other signaling pathways (Figure 1h–j). These findings suggest that, due to the major variation in TGF‐β signaling pathway regulation during the development of ectodermal and endodermal epithelia, adopting PRM for EEO fabrication leads to over enhancement of the TGF‐β signaling pathway and EMT, which further results in poor recapitulation of native ectodermal epithelial characteristics (Figure 1k). Therefore, we inferred that downregulation of the TGF‐β signaling pathway could improve the fabrication of EEOs.

### 2.2 Downregulation of TGF‐β Signaling Pathway Suppresses EMT and Enhances Ectodermal Epithelial Properties of EEOs

**FIGURE 2:**
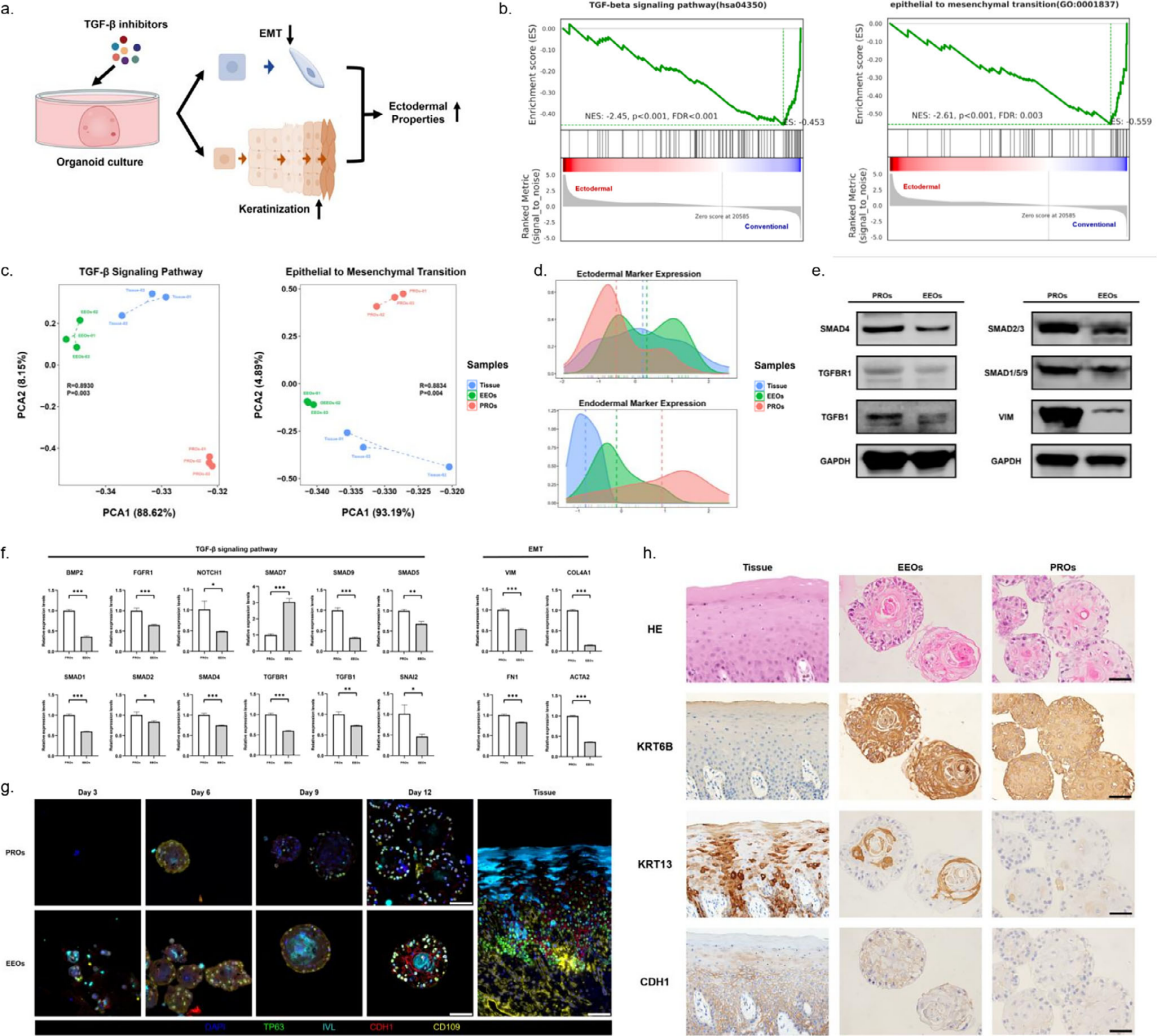
Downregulation of TGF‐β signaling pathway suppresses EMT and enhances the ectodermal epithelial properties of EEOs. a) Schematic diagram of the proposed strategy for EEOs fabrication. b) GSEA analysis of DEGs between EEOs and PROs showing significant downregulation of the TGF‐β signaling pathway and EMT in EEOs. c) PCA analysis depicting the correlation of gene expression patterns related to the TGF‐β signaling pathway and EMT between oral epithelial tissues, EEOs, and PROs. d) Density plots depicting the expression levels of ectodermal marker genes in oral epithelial tissues, EEOs, and PROs. e) Representative western blotting images comparing expression levels of critical markers related to the TGF‐β signaling pathway and EMT between EEOs and PROs. f) qRT‐PCR results comparing relative expression levels of critical genes related to the TGF‐β signaling pathway and EMT between EEOs and PROs (data are shown as means ± SEM; t‐test; n = 3). g) Multi‐label fluorescence assays demonstrating expression patterns of TP63, IVL, CDH1, and CD109 between oral epithelial tissues, EEOs, and PROs (bar = 50 µm). h) Representative H&E and IHC images of oral epithelial tissues, EEOs, and PROs, comparing the expression of differentiation marker KRT6B, cornification marker KRT13, and ectodermal epithelium marker CDH1 (bar = 100 µm). *p <0.05, **p < 0.01, and ***p < 0.001. Statistical significance was set at p < 0.05.

Based on the notion of modeling development, a new strategy for EEOs fabrication that could faithfully recapitulate the regulation of crucial developmental patterns of ectodermal epithelia required development [27, 28]. Referring to the divergence between ectodermal and endodermal epithelium development, along with the variations between PROs and native ectodermal epithelia, EEO establishment was carried out with more attention to the regulation of TGF‐β signaling pathway. Specifically, we examined whether downregulating the TGF‐β signaling pathway could suppress EMT and thereby facilitate the development of EEOs (Figure 2a). Oral epithelial tissues dissected from the patients during surgery were dissociated into single cells and resuspended in Matrigel. The culture medium was modified by supplementing it with SB505124, ITS‐X, hydrocortisone, and Wnt‐3A. The organoids formed spherical structures on day 3 of culture and gradually reached a mature state on day 12.

To verify the effectiveness of our strategy, RNA‐sequencing assays were conducted to compare the molecular features of oral epithelial tissues, EEOs, and PROs. Differential enrichment analysis and GSEA analysis revealed a significant downregulation of TGF‐β signaling pathway and EMT in EEOs, compared with PROs (Figure 2b). Besides, BMP signaling pathway, which is closely related to TGF‐β signaling pathway and EMT, was also significantly downregulated in EEOs (Figure S2a). Regarding the expression patterns of genes related to the TGF‐β signaling pathway and EMT, principal component analysis (PCA) revealed that our new strategy markedly improved the similarity between EEOs and epithelial tissues (Figure 2c). Consequently, EEOs possessed higher levels of ectodermal marker gene expression and lower levels of endodermal gene expression than PROs, consistent with oral epithelial tissues (Figure 2d). Clustering analysis further demonstrated that regarding the expression patterns of germ layer specific genes EEOs shared greater similarity with oral epithelial tissues, compared to that between PROs and oral epithelial tissues (Figure S2b). Western blotting assays verified the reduced expression of TGF‐β receptors, ligands, and intracellular signal transducer proteins (SMAD family members) in EEOs, as compared with PROs (Figure 2e; Figure S2c). Immunocytochemical (ICC) and qRT‐PCR assays of the expression levels of critical markers including *TGFBR1*, *FGFR1*, *SMAD1/5/9*, *SMAD2/3*, *TGFB1*, *CDH1*, *LUM*, and *VIM* confirmed the synchronous downregulation of the TGF‐β signaling pathway and EMT in EEOs (Figure 2f; Figure S2d,e).

To validate the impact of TGF‐β signaling pathway and EMT downregulation on EEO fabrication, we examined the expression of critical proteins related to ectodermal epithelial development. Multi‐label fluorescence assays on days 3, 6, 9, and 12 of organoid culture confirmed that EEOs and oral epithelial tissues expressed significantly higher levels of the TGF‐β signaling pathway antagonist CD109 and ectodermal epithelium‐specific marker CDH1, compared with PROs [29, 30]. TP63 and IVL, both regarded as crucial markers for ectodermal epithelial cell differentiation, were also highly expressed in EEOs, similar to their expression in oral epithelial tissues (Figure 2g) [31, 32]. In terms of recapitulating ectodermal epithelial properties in the mature state, namely on day 12 of culture, immunohistochemistry (IHC) analysis revealed that the keratinocyte differentiation marker KRT‐6B and the epithelial adhesion marker CDH1, both of which are critical hallmarks of the ectodermal epithelium, were expressed at significantly higher levels in EEOs than in PROs. Notably, EEOs possessed a distinct section of KRT‐13^+^ flattened enucleated cells that closely resembled the terminally differentiated keratinocytes in ectodermal epithelial tissues (Figure 2h) [33]. Hence, downregulation of the TGF‐β signaling pathway significantly suppressed EMT and enhanced the ectodermal epithelial properties of EEOs by modeling the natural developmental process.

### 2.3 The Effect of TGF‐β Signaling Downregulation on the Capacity of EEOs to Recapitulate Ectodermal Epithelial Barrier Function

**FIGURE 3:**
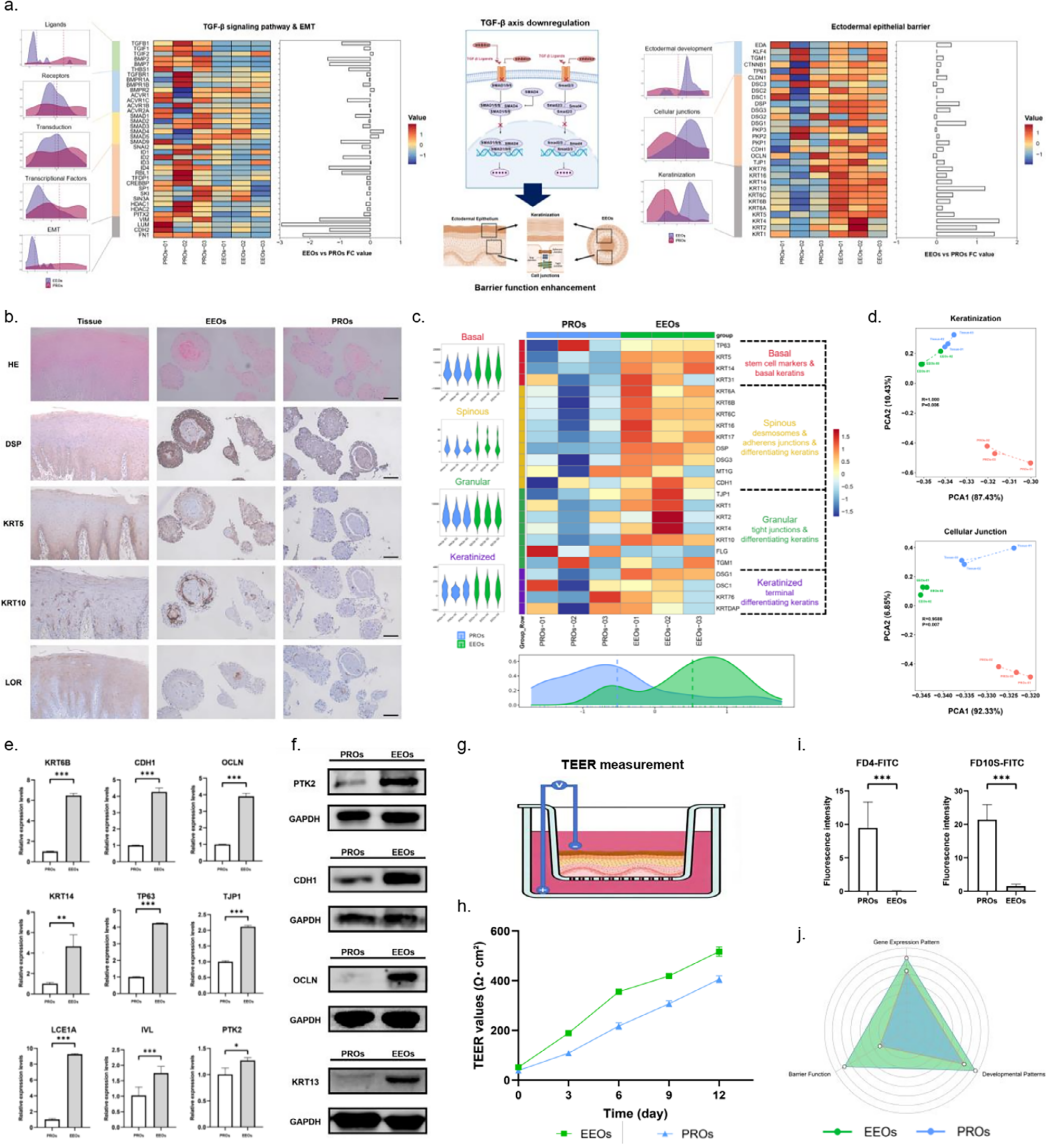
Downregulation of TGF‐β signaling pathway enhances organoids’ capacity to recapitulate the barrier function of ectodermal epithelial tissues. a) Combined density plot, heatmap, and bar plot depicting expression patterns of genes related to TGF‐β signaling pathway and genes related to ectodermal epithelial barrier function between EEOs and PROs. b) Representative H&E and IHC images of oral epithelial tissues, EEOs, and PROs, comparing the expression of stem cell marker KRT5, spinous cell marker DSP, granular cell marker KRT10, and cornified marker LORICRIN (bar = 100 µm). c) Combined density, violin, and bar plots depicting the expression patterns of genes related to keratinocyte subtype markers between EEOs and PROs. d) PCA depicting correlations among the expression patterns of genes related to keratinization and cellular junctions between oral epithelial tissues, EEOs, and PROs. e) qRT‐PCR analysis comparing the relative expression levels of critical genes related to ectodermal epithelial barrier function between EEOs and PROs (data are shown as means ± SEM; t‐test; n = 3). f) Representative western blot images comparing the expression levels of critical markers related to ectodermal epithelial barrier function between EEOs and PROs. g) Schematic diagram of TEER measurement assays. h) Line chart depicting divergence in TEER between EEOs and PROs (data are shown as means ± SEM; t‐test; n = 3). i) Quantification of FD4‐FITC and FD10S‐FITC fluorescence permeability assays comparing barrier integrity between EEOs and PROs (data are shown as means ± SEM; t‐test; n = 3). j) Radar plot depicting the superiority of EEOs over PROs in recapitulating epithelial barrier function. *p <0.05, **p < 0.01, and ***p < 0.001. Statistical significance was set at p < 0.05.

The barrier function, represented by the formation of the stratum corneum and cellular junctions, is the primary function of the ectodermal epithelia (Figure 3a). Since the downregulation of the TGF‐β signaling pathway significantly enhanced the ectodermal epithelial properties of EEOs, we further examined whether it could also enhance the organoids’ capacity to recapitulate the barrier functions of ectodermal epithelial tissues. Differential enrichment analysis between the gene expression patterns of EEOs and PROs demonstrated that while genes related to the TGF‐β signaling pathway were significantly downregulated in EEOs, both ectodermal developmental markers and barrier function markers were significantly upregulated in EEOs (Figure 3a). H&E staining of mature organoids demonstrated that EEOs recapitulated the tight structural arrangement of keratinocytes, similar to the hierarchical arrangement of cells found within epithelial tissues in vivo. A gradual transition from cubic basal cells to flat and enucleated keratinized cells is well represented in EEOs [34, 35]. IHC analysis comparing the expression levels of keratinocyte subtype protein markers revealed that EEOs possessed significantly higher expression levels of the basal cell marker KRT5, spinous cell marker DSP, granular cell marker KRT10, and keratinized cell marker LORICRIN than PROs. Moreover, the hierarchical configuration of keratinocyte subtype protein markers in EEOs closely resembled that in native epithelial tissues (Figure 3b). Clustering analysis also indicated that EEOs possessed higher expression levels of typical keratinocyte subtype markers than PROs (Figure 3c). In addition, EEOs expressed higher levels of KRT2E, KRT4, and KRT6B, which are regarded as typical markers for the formation of spinous and granular layers, than PROs throughout the culture process Figure S3a,b) [36, 37, 38]. Hence, we deduced that our new ectodermal organoid fabrication strategy effectively facilitated the committed differentiation of keratinocytes and thus improved the EEOs’ cell subtype and structural representation of ectodermal epithelial tissues.

The stratum corneum and cellular junctions are regarded as the structural bases of the ectodermal epithelial barrier. PCA of the expression patterns of genes related to keratinization and cellular junctions indicated that the similarity between EEOs and oral epithelial tissues was markedly higher than that between PROs and epithelial tissues (Figure 3d) [39, 40]. qRT‐PCR and western blotting assays confirmed that ectodermal epithelium‐specific markers, along with genes related to ectodermal epithelium development, such as *CDH1*, *IVL*, *LCE1A, TJP1*, *OCLN*, *PTK2*, and *TP63*, were significantly upregulated in EEOs (Figure 3e,f; Figure S3c,d). ICC analysis revealed that EEOs possessed significantly higher expression levels of cellular junction and keratinization protein markers, including DSP, DSG3, TJP1, KRT1, KRT10, KRT13, FLG, and CD109 than PROs (Figure S3e,f). In terms of the overall similarity of gene expression patterns, differential enrichment analysis revealed a reduced count of differentially expressed genes (DEGs) between EEOs and oral epithelial tissues compared to that between PROs and oral epithelial tissues (Figure S3g). Pearson's correlation analysis indicated a narrow divergence in gene expression patterns between EEOs and oral epithelial tissues (Figure S3h).

To validate whether EEOs could recapitulate ectodermal epithelial barrier function, trans‐epithelial electrical resistance (TEER) and barrier permeability assays were conducted. Throughout the 12 d culture period, EEOs possessed a significantly higher TEER than the PROs (Figure 3g,h). Barrier permeability assays carried out using FD4 and FD10S fluorescein isothiocyanate‐dextran further demonstrated that EEOs significantly reduced the permeability of the epithelial barrier compared to PROs (Figure 3i). Thus, we concluded that the barrier integrity of EEOs is superior to that of PROs. Moreover, EEOs possessed more distinct populations of KRT13^+^ enucleated keratinocytes than PROs, indicating that our strategy strengthened the formation of the stratum corneum (Figure S3i) [41]. In summary, this strategy significantly enhanced organoids’ capacity to recapitulate the barrier functions of ectodermal epithelial tissues (Figure 3j).

**FIGURE 4:**
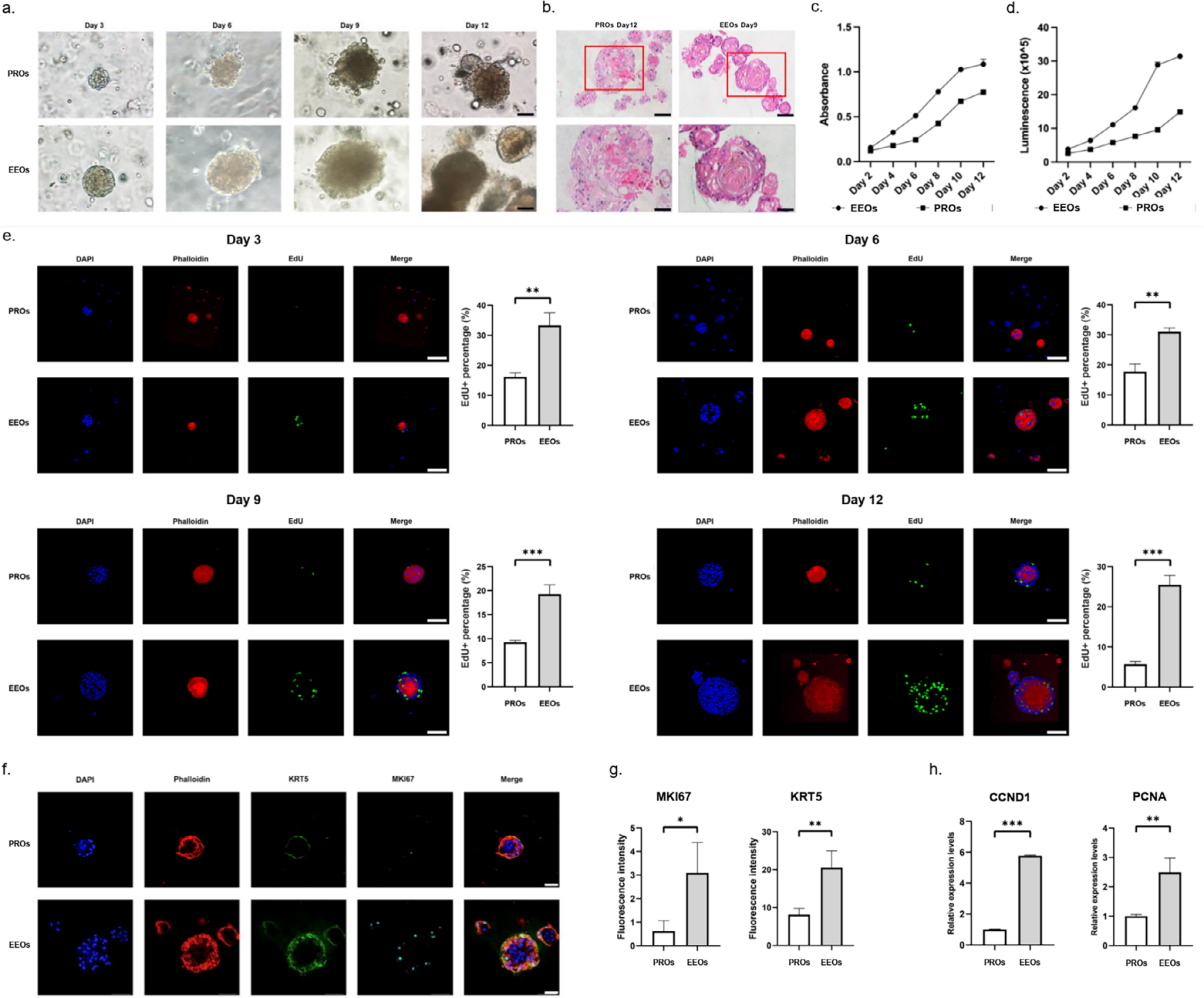
Enhanced fabrication efficiency of EEOs through modeling development. a) Light microscopy observations of EEOs and PROs on days 3, 6, 9, and 12 of culture (bar = 100 µm). b) Representative H&E images of EEOs and PROs on days 9 and 12 of culture, respectively (bar = 100 µm/50 µm). c) Line chart depicting divergence in CCK‐3D analysis between EEOs and PROs. d) Line chart depicting the divergence in ATP synthesis between EEOs and PROs. e) Representative EdU immunofluorescence images and quantitation on days 3, 6, 9, and 12, which compare the percentages of active proliferating cells between EEOs and PROs (bar = 100 µm). f) Representative ICC images comparing the expression of proliferation marker MKI67 and stem cell marker KRT5 between EEOs and PROs (bar = 100 µm). g) ICC quantitation of proliferation marker MKI67 and stem cell marker KRT5 between EEOs and PROs (data are shown as means ± SEM; t‐test; n = 4). h) qRT‐PCR analysis of the relative expression levels of critical genes related to the proliferation activity between EEOs and PROs (data are presented as means ± SEM; t‐test; n = 3). *p <0.05, **p < 0.01, and ***p < 0.001. Statistical significance was set at p < 0.05.

The skin and oral mucosa both originate from the ectoderm and share similar cellular and histological features [34, 40]. Therefore, this strategy was extended to the establishment of epidermal organoids. Dorsal epithelial tissues dissected from the rats were dissociated into single cells and resuspended in Matrigel. Organoid culture was then performed in both the new ectodermal‐specific medium and PRM. Compared to epidermal organoids established by PRM, organoids established by ectodermal‐specific medium possessed a higher growth rate, which was characterized by larger mean diameters on day 12 of culture, along with greater numbers of organoids (Figure S4a). With regard to histological structures, epidermal organoids established using ectodermal‐specific medium possessed a clearly visible layer of the stratum corneum, which was significantly more obvious than that of epidermal organoids established using PRM (Figure S4b). Epidermal organoids established using an ectodermal‐specific medium also showed significantly higher expression levels of epidermal developmental markers KRT2E, KRT6B, and CTNNB1 (Figure 4c–f). In summary, our new ectodermal‐specific medium is capable of increasing the growth rate and promoting the development of epidermal organoids.

### 2.4 Enhanced Fabrication Efficiency of EEOs through Modeling Development

A prolonged culture period is a major hurdle to the generalization of organoid applications [42, 43]. Therefore, we investigated whether our strategy could enhance the efficiency of EEOs fabrication. Light microscopy observations revealed that under the same culture period, the mean diameter of EEOs significantly exceeded that of PROs, suggesting that EEOs possessed a higher growth rate (Figure 4a). Notably, EEOs developed into stratified structures with a distinct stratum corneum within 8–9 d. In comparison, PROs took 12–14 d to reach a mature state, which is consistent with previous studies (Figure 4b) [17, 18]. CCK‐3D cell proliferation and ATP synthesis assays both confirmed that EEOs displayed a significantly higher growth rate than PROs (Figure 4c,d). EdU quantitative assays demonstrated a significantly increased percentage of propagating cells in EEOs throughout the culture process compared with that in PROs (Figure 4e). ICC assays revealed that both the proliferation marker MKI‐67 and stem cell marker KRT‐5 were expressed at significantly higher levels in EEOs (Figure 4f,g). Moreover, qRT‐PCR confirmed the higher expression levels of the proliferation markers *CCND1* and *PCNA* in EEOs than in PROs (Figure 4h). These results suggest that the high culture efficiency of EEOs stems primarily from their enrichment in highly proliferative ectodermal epithelial cells. Notably, when applying our ectodermal epithelial‐specific culture medium, the outgrowth efficiency of the organoids reached up to 90%, which was higher than that previously reported [18]. Hence, by markedly shortening the culture period of EEOs, their widespread application could be facilitated.

### 2.5 EEOs Can Detect the Damaging Effects of Nanomaterials on Ectodermal Epithelial Cell Junctions

**FIGURE 5:**
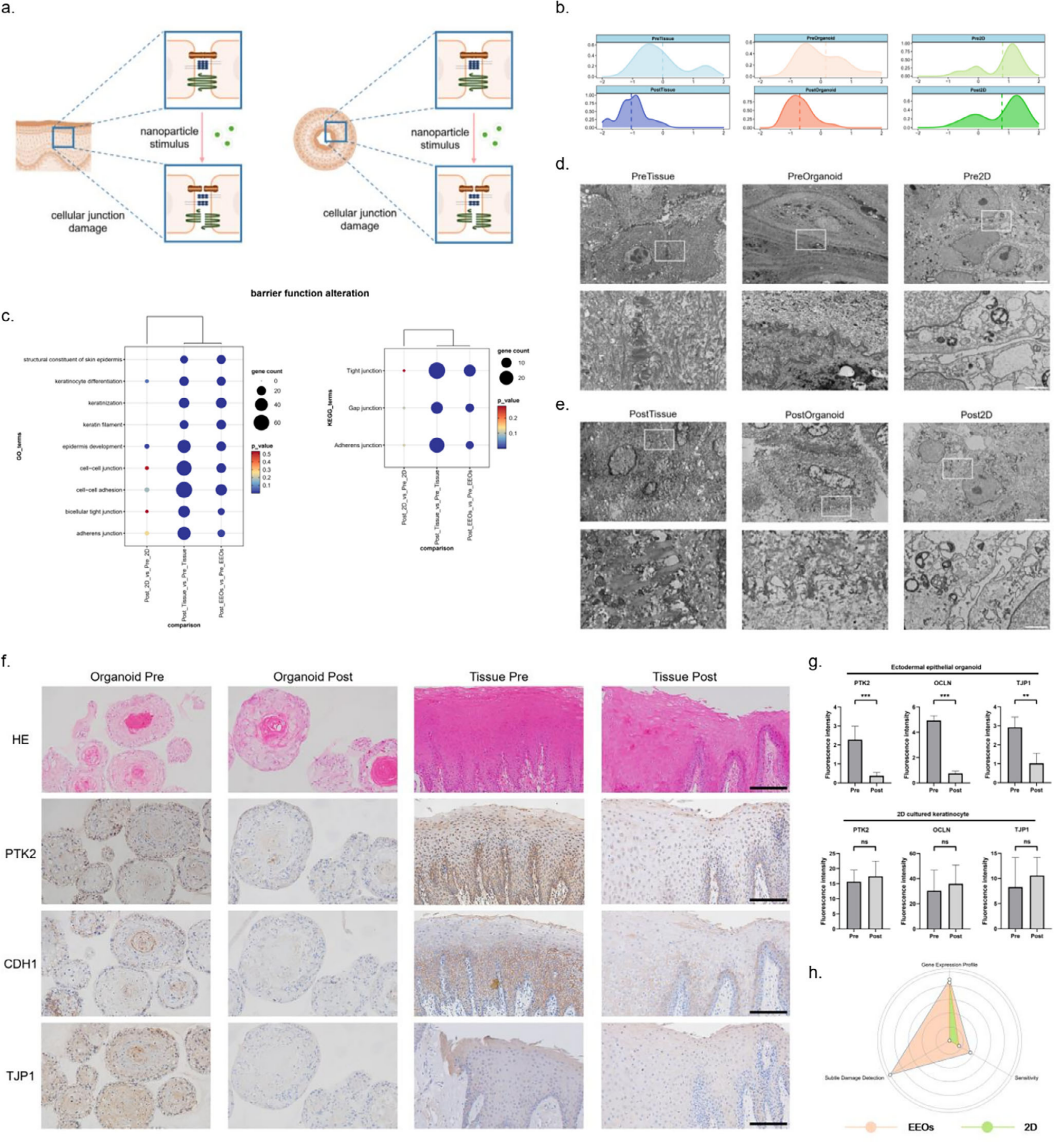
EEOs can sensitively detect the damaging effect of nanomaterials on ectodermal epithelial cell junctions. a) Schematic diagram depicting the capability of EEOs to recapitulate the damaging effects of fluorescent nanoprobes on epithelial cell junctions. b) Density plot and clustering heatmap depicting alterations in the expression levels of critical markers related to cellular junctions. c) Enrichment factor diagram depicting GO and KEGG analyses of DEGs, comparing the enrichment of terms related to cellular junctions and epithelial barrier functions. d) Representative TEM images depicting cellular junction and actin filament structures of epithelial tissues, EEOs, and 2D cultured keratinocytes prior to AA nanoprobe stimuli (bar = 5µm; 1 µm). e) Representative images of TEM depicting the cellular junction and actin filament structures of epithelial tissues, EEOs, and 2D cultured keratinocytes after AA nanoprobe exposure (bar = 5 µm; 1 µm). f) Representative H&E and IHC images of EEOs and epithelial tissues exposed to AA nanoprobes, comparing alterations in the expression of cellular junction proteins PTK2, CDH1, and TJP1 (bar = 100 µm). g) ICC quantitation of the cellular junction markers PTK2, OCLN, and TJP1 (data are shown as means ± SEM; t‐test; n = 4). h) Radar plot depicting the superiority of EEOs over 2D cultured keratinocytes in assessing the damaging effects of fluorescent nanoprobes on epithelial cell junctions. *p <0.05, **p < 0.01, and ***p < 0.001. Statistical significance was set at p < 0.05.

Evaluating the impact of pharmaceuticals and biomaterials on the critical structures of the ectodermal epithelial barrier, especially cellular junctions, is crucial for pre‐clinical assessments [4, 44, 45, 46, 47, 48]. Considering the preeminent capacity of EEOs to represent the barrier functions of the ectodermal epithelium, we sought to explore whether EEOs could serve as a potential platform for epithelial barrier stimulation assessments of pharmaceutics and biomaterials. Currently, 2D keratinocytes are most used in pre‐clinical assessments [49]. However, their sensitivities and accuracies are far from ideal. To comprehensively represent divergent categories of nanoparticles, we used acrylic acid (AA) neutral red‐fluorescent nanoprobes as an example of small‐molecule monomer nanomaterials and polymethyl methacrylate (PMMA) particles as an example of high‐molecular polymer nanoparticles. The former has a mean diameter of 4 nm, whereas the latter has a mean diameter of 100 nm. Thus, our screening assays covered both minor‐ and major‐scale nanoparticles. We compared the sensitivity of detecting subtle damage to the epithelial barrier between EEOs and 2D cultured keratinocytes (Figure 5a; Figure S5a).

We applied 2 mg/mL AA nanoprobes to the culture medium for 4 h to ensure that they could fully penetrate 2D keratinocytes and EEOs, and investigated the sensitivity of detecting alterations in gene expression patterns caused by nanoprobe stimulation (Figure S5b). RNA sequencing detected many DEGs before and after AA stimulation in both epithelial tissues and EEOs, yet not in 2D cultured keratinocytes (Figure S5c). Notably, RNA sequencing revealed a drastic downregulation of gene expression related to cellular junctions, such as *TJP1*, *CDH1*, and *DSG2*, in epithelial tissues and EEOs after AA stimulation by differential enrichment analysis. No significant variation was detected in 2D cultured keratinocytes (Figure 5b; Figure S5d). GO enrichment analysis of DEGs before and after AA nanoprobe exposure further confirmed that EEOs could recapitulate the significant cellular junctions impairments caused by AA nanoprobes in epithelial tissues. In contrast, no significant variation in cellular junctions was detected in 2D cultured keratinocytes before and after AA nanoprobe exposure (Figure 5c). Thus, we demonstrate that EEOs are superior to 2D cultured keratinocytes in recapitulating alterations in gene expression, especially those of cellular junction marker genes, caused by AA stimulation.

We investigated the damaging effects of nanoparticles on the epithelial barrier. Transmission electron microscopy (TEM) revealed that EEOs closely recapitulated the subcellular structure of cellular junctions and actin filaments in epithelial tissues (Figure 5d). Furthermore, the apparent disruption of these structures caused by AA nanoprobes was detected in EEOs and epithelial tissues using TEM. In contrast, these subcellular structures were not represented in 2D cultured keratinocytes (Figure 5e). Histological examination revealed that the expression of critical cellular junction protein markers, including PTK2, CDH1, and TJP1, in the oral epithelial tissue and EEOs was markedly downregulated after exposure to AA nanoprobes (Figure 5f) [4, 50, 51]. Western blotting demonstrated that oral epithelial tissues and EEOs possessed similar levels of cell junction protein expression, both of which were significantly higher than that in 2D cultured keratinocytes (Figure S5e). ICC assays and quantitative analysis also confirmed that the expression levels of PTK2, OCLN, and TJP1 were significantly decreased in EEOs after AA nanoprobe exposure, whereas no significant alterations were detected in 2D cultured keratinocytes (Figure 5g; Figure S5f,g).

Furthermore, we assessed the ability of EEOs to detect the toxicological effects of PMMA nanoparticles, a representative high‐molecular‐weight polymer nanomaterial. After exposure to 2 mg/mL PMMA nanoparticles for 4 h, qRT‐PCR detected significant downregulation of the expression levels of the barrier function markers *CDH1*, *KRT13*, and *PTK2* in both EEOs and oral epithelial tissues. In contrast, no significant alteration in barrier function marker expression was detected in 2D cultured keratinocytes (Figure S5h). ICC assays further confirmed that EEOs were capable of sensitively detecting the downregulation of CDH1, KRT13, and PTK2 induced by PMMA stimulation (Figure S5i).

In summary, EEOs can sensitively detect the subtle damage caused by AA nanoprobes at the cellular junctions of epithelial tissues. In addition, in terms of recapitulating the alterations in gene expression patterns, subcellular structures, and barrier functions of epithelial tissues after nanoprobe stimulation, EEOs were significantly superior to 2D cultured keratinocytes. Thus, EEOs may serve as a reliable platform for assessing the potential damage caused by nanomaterials to ectodermal epithelial barrier functions (Figure 5j).

### 2.6 EEOs Can Closely Recapitulate the Toxic Effect of Pharmaceutics on Ectodermal Epithelium

**FIGURE 6:**
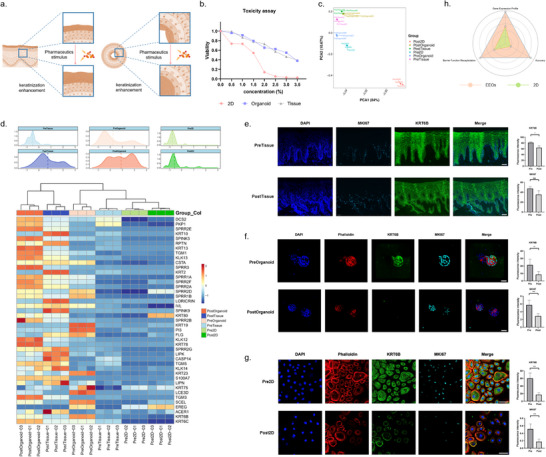
EEOs can closely recapitulate the toxic effect of pharmaceutics on ectodermal epithelium. a) Schematic diagram illustrating the ability of EEOs to assess the effects of pharmaceuticals on epithelial tissues. b) Line chart depicting the toxic effect of Listerine on oral epithelial tissues, EEOs, and 2D cultured keratinocytes; cell viability is plotted on the y‐axis for different concentrations of Listerine, ranging from 0% to 3.5% (v/v; x‐axis). c) PCA depicting the correlation of gene expression profiles between oral epithelial tissues, EEOs, and 2D cultured keratinocytes before and after Listerine stimulation. d) Density plot and clustering heatmap depicting the alterations in cornification‐related gene expression in oral epithelial tissues, EEOs, and 2D cultured keratinocytes induced by Listerine stimulation. e) Representative ICC staining images and quantitation of oral epithelial tissues before and after Listerine stimulation, comparing alterations in the expression levels of differentiation marker KRT6B and proliferation marker MKI67 (bar = 100 µm, data are shown as means ± SEM: t‐test; n = 3). f) Representative ICC staining images and quantitation of EEOs before and after Listerine stimulation, comparing alterations in the expression levels of differentiation marker KRT6B and proliferation marker MKI67 (bar = 50 µm, data are shown as means ± SEM; t‐test; n = 3). g) Representative ICC staining images and quantitation of 2D cultured keratinocytes before and after Listerine stimulation, comparing alterations in the expression levels of differentiation marker KRT6B and proliferation marker MKI67 (bar = 20 µm; data are shown as means ± SEM; t‐test; n = 3). h) Radar plot depicting the superiority of EEOs over 2D cultured keratinocytes in assessing the toxicological effects of Listerine on oral epithelial tissues. *p <0.05, **p < 0.01, and ***p < 0.001. Statistical significance was set at p <0.05.

In addition to detecting the potentially damaging effects of nanomaterials and pharmaceuticals on epithelial tissues, viability assays are also crucial during pre‐clinical screening. Thus, we performed pharmaceutical stimulation assays to compare the accuracy of the currently utilized 2D cultured keratinocytes and EEOs. Listerine (a commercially available mouthwash) was used for stimulation assays because it is extensively applied in oral hygiene maintenance, which represents a wide category of topical agents. Different concentrations of Listerine ranging from 0.5% to 3.5% (v/v) were applied as external stimuli to the oral epithelial tissues, EEOs, and 2D cultured keratinocytes (Figure 6a; Figure S6a). EdU assays showed that EEOs closely mimicked the toxic effects of Listerine on oral epithelial tissues. In contrast, 2D cultured keratinocytes were hypersensitive to Listerine stimulation (Figure 6b; Section S1). Live/dead staining and Tunel assays further confirmed that EEOs could accurately assess the toxic effects of Listerine on epithelial tissues, as compared with 2D keratinocytes (Section S2). Hence, EEOs may significantly improve the accuracy of viability assays. Furthermore, RNA‐Seq assays revealed a slight divergence in gene expression patterns between EEOs and oral epithelial tissues after stimulation. In contrast, 2D cultured keratinocytes possessed apparently divergent gene expression patterns compared to epithelial tissues after stimulation (Figure 6c; Figure S6b). In addition, the number of DEGs between EEOs and epithelial tissues was significantly lower than that between 2D cultured keratinocytes and epithelial tissue, both before and after Listerine stimulation (Figure S6c). Then, the alterations of gene expressions related to critical signaling pathways including TGF‐β, TNF, and NF‐κB were examined. GSEA revealed that EEOs possessed alterations consistent with epithelial tissues, while 2D cultured keratinocytes displayed opposite alterations (Figure S6d). These findings suggest that EEOs can more accurately assess the alterations in viability and gene expression of ectodermal epithelial tissues in response to Listerine stimulation, as compared to 2D cultured keratinocytes.

We further examined the effect of Listerine stimulation on epithelial barrier function. The stratum corneum, a pivotal structure in the ectodermal epithelial barrier, plays an indispensable role in resisting external stimuli [1, 35]. IHC assays demonstrated that EEOs share strong similarities in cell type and structure with epithelial tissues in response to Listerine. Notably, TP63+ stem cells and MKI67+ proliferating cells were located in the peripheral region of the organoids, while KRT13+‐enucleated keratinocytes were located in the center. In contrast, the monolayer‐cultured keratinocytes were homogeneous in terms of cellular subtypes and structure (Figure S6e). GSEA–GO analysis of DEGs between EEOs and 2D‐cultured keratinocytes revealed that biological functions related to barrier function were significantly upregulated in EEOs (Figure S6f). Notably, after Listerine stimulation, clustering analysis revealed that EEOs were capable of significantly upregulating cornification markers in epithelial tissues, which was not observed in 2D cultured keratinocytes (Figure 6d). Due to the protection of the stratum corneum, no significant variation in proliferation and apoptosis marker genes was detected in the epithelial tissues and EEOs after Listerine stimulation. In contrast, 2D cultured keratinocytes underwent drastic downregulation of proliferation markers and significant upregulation of apoptosis markers after Listerine stimulation (Figure S6g,h). ICC assays and quantitative analysis of the expression levels of the proliferation marker MKI67 and the differentiation marker KRT6B further validated similar alterations in biological processes within EEOs and oral epithelial tissues. In contrast, the expression levels of both markers decreased dramatically after stimulation in 2D cultured keratinocytes, indicating an exacerbated biological response to stimulation (Figure 6e–g; Section S3). Listerine stimulation did not induce significant morphological alterations in epithelial tissues or EEOs, whereas 2D cultured keratinocytes gradually lost their original morphology (Section S4). These findings elucidate why 2D cultured keratinocytes are hypersensitive to Listerine.

In summary, conventional 2D cultured keratinocytes are more hypersensitive to external stimuli than epithelial tissues, which may lead to false‐positive results in drug toxicity screening. Therefore, EEOs may serve as accurate platforms for pre‐clinical toxicity screening (Figure 6h).

## 3 Discussion

In vitro biomimetic models are of great significance in developmental, toxicological, and regenerative medical research. At present, the pre‐clinical evaluation of drugs and nanomaterials faces great challenges owing to the lack of a satisfactory screening platform [46, 52]. According to our study, the core principles for establishing in vitro models include three major aspects. First, biomimetic models should closely represent the developmental processes in native tissues or organs. Second, the molecular, cellular, and structural characteristics of mature tissues should be simulated using in vitro models. Third, biomimetic models should be capable of recapitulating the primary functions of the tissues and organs. In this study, we fabricated EEOs by referring to the natural developmental patterns of ectodermal epithelia. We demonstrated that downregulation of the TGF‐β signaling pathway during EEOs fabrication plays a pivotal role in cell fate determination and the committed differentiation of ectodermal epithelial stem cells. Consequently, keratinocytes differentiate into specific subtypes and form hierarchical structures identical to those of native epithelial tissues, which are fundamental for simulating the biological response of the ectodermal epithelial barrier to external stimuli. We demonstrated that subtle damage caused by nanomaterials to the cellular junctions of the epithelial barrier, which had been previously overlooked, could be sensitively detected by EEOs. Moreover, the EEOs in this study precisely recapitulated the effects of Listerine on viability and the gene expression pattern of the oral mucosa. To the best of our knowledge, this is the first ectodermal epithelial organoid‐based assessment platform for barrier stimulation testing of pharmaceuticals and biomaterials.

Studies on embryonic development indicate that the regulatory patterns of the TGF‐β signaling pathway vary significantly across germ layers. Its upregulation promotes endodermal epithelial development (e.g., gastrointestinal tissues), whereas its downregulation is essential for ectodermal lineage commitment [24, 53, 54, 55]. The findings of this study confirm that among the regulatory patterns of crucial pathways related to tissue development, the TGF‐β signaling pathway shows the greatest divergence between ectodermal and endodermal epithelia. Moreover, the TGF‐β signaling pathway plays a fundamental role in regulating EMT during both tissue development and tumorigenesis [53, 55, 56, 57]. We further verify that markers related to both the TGF‐β signaling pathway and EMT are significantly upregulated in the PROs compared to native ectodermal epithelia. Hence, we conclude that excessive EMT impedes the committed differentiation of critical epithelial cell subtypes and thereby restricts the ability of PROs to recapitulate the barrier function of ectodermal epithelia. Based on this paradigm, we implemented an innovative strategy for EEOs fabrication by targeted downregulation of the TGF‐β signaling pathway. Our approach significantly inhibited EMT during EEOs development and successfully recapitulated key features of the ectodermal epithelium, including the formation of a stratified structure containing KRT13^+^ keratinized cells, TP63^+^ stem cells, and functional cellular junctions (CDH1, DSP, and TJP1), all of which are critical for barrier function [4, 40, 41, 50]. The findings of this study underscore the importance of tailoring organoid culture conditions according to germ layer‐specific developmental principles, thereby providing a framework for engineering more physiologically relevant tissue models.

Pre‐clinical toxicity screening is an essential procedure in the development of innovative pharmaceuticals and biomaterials [58, 59, 60, 61, 62, 63, 64, 65]. However, owing to the lack of accurate and sensitive platforms, current methods for pre‐clinical screening have fallen behind [66]. In this study, we demonstrated that EEOs provide an ideal platform for assessing the effects of pharmaceuticals and biomaterials on the ectodermal epithelium. Compared to the widely utilized 2D cultured cell lines, EEOs represent the stratified structure of the ectodermal epithelia. As a result, the process of pharmaceuticals and biomaterials penetrating the epithelial barrier and their effects on barrier function can only be simulated using EEOs [67]. In other words, 2D cultured keratinocyte‐based platforms can only assess the effects of pharmaceuticals and biomaterials at the cytological level, whereas EEO‐based platforms extend assessments to the histological level. Therefore, the use of EEOs in pre‐clinical screening can significantly improve the accuracy of dose‐limiting toxicity assessments, which may broaden the application of certain pharmaceutics and biomaterials [68]. Moreover, because EEOs can faithfully recapitulate the alterations in gene and protein expression induced by pharmaceuticals and biomaterials, they may provide significant insights into the examination of critical biological mechanisms in pre‐clinical screening [12, 47, 64, 69, 70, 71].

It is generally held that the ectodermal epithelium serves as the primary immunological barrier of our body [1, 34, 39, 40]. In addition to cell viability, the structural and functional integrity of cellular junctions are important for protection against microbial invasion and pathogen penetration [4, 39]. In this study, EEOs showed superior sensitivity in detecting compromised junctional integrity induced by AA fluorescent nanoparticles. This enhanced detection capability stems from the remarkable capacity of EEOs to faithfully reconstruct the native architecture and molecular composition of the intercellular junctions, which are characteristic of ectodermal epithelia. To our knowledge, this is the first study of EEOs that clearly depicts the subcellular structure of the epithelial barrier. Notably, conventional 2D keratinocyte cultures fail to detect alterations in junction‐related gene expression or protein distribution patterns under identical experimental conditions. Considering the ubiquitous application of topical formulations, including cosmetic products, dental biomaterials, and transdermal therapeutics, the implementation of EEO‐based pre‐clinical screening platforms could substantially mitigate infection risks associated with compromised barrier function.

This study presents a highly reproducible and scalable organoid‐based screening platform for pre‐clinical assessment. However, deficiencies remain in the establishment and validation of the EEOs model. In this study, the molecular characteristics of the organoids and epithelial tissues were verified using bulk‐RNA sequencing. With regard to the cellular heterogeneity of ectodermal epithelial tissues and organoids, single‐cell RNA sequencing may facilitate more elaborate examinations. Regarding the toxicological screening of nanomaterials, our study covered two of the most commonly applied categories of nanoparticles: minor‐diameter nanoprobes and major‐diameter prosthetic nanomaterials. However, our study did not cover the toxicity assessments of nanoparticles with varying surface charges or nanomaterials with other compositions. Thus, further exploration of diverse nanomaterials is required to strengthen the clinical significance of organoid‐based toxicological screening. In addition, examination of the chronic toxicological effects of topical agents or biomaterials requires organoids capable of long‐term cultivation, which was not performed in the current study. Further research on culture conditions committed to the long‐term maintenance of EEOs is essential for the detection of chronic toxicological effects.

## 4 Conclusions

In summary, we proposed an innovative EEO‐based platform for assessing the effects of pharmaceuticals and nanomaterials on the epithelial barrier. We significantly improved the capability of organoids to represent the cell subtypes, structures, and barrier functions of the ectodermal epithelium by downregulating the TGF‐β signaling pathway. Furthermore, we confirmed that our EEO‐based screening platform remarkably improves the accuracy and sensitivity of epithelial barrier stimulation assessments. The findings address a longstanding gap in pre‐clinical screening protocols, where the assessment of the ectodermal epithelial barrier has been consistently overlooked. The EEOs platform not only provides a more sensitive tool for barrier function evaluation but also offers unprecedented mechanistic insights into the regulation of ectodermal epithelial barrier properties at the molecular, cellular, and tissue levels.

## Funding

This research was supported by the National Key R&D Program of China (2022YFA1207304), Beijing Natural Science Foundation (L242154), Beijing Research Ward Excellence Program (BRWEP2024W 194100100) and Clinical Research Foundation of Peking University School and Hospital of Stomatology (PKUSS‐2024CRFG02).

## Conflicts of Interest

The authors declare no conflict of interest.

## Supporting information




**Supporting File**: advs75041‐sup‐0001‐SuppMat.docx.

## Data Availability

The data that support the findings of this study are available from the corresponding author upon reasonable request.
