# Supplementary material for: Highly Biomimetic Ectodermal Epithelial Organoids for Epithelial Barrier Stimulation Assays
Source: Adv Sci (Weinh). 2026 Mar 28;13(39):e22709. doi: 10.1002/advs.202522709 (PMC13325922; doi:10.1002/advs.202522709)
Supplement: Supplementary file 1 — Supporting File: advs75041‐sup‐0001‐SuppMat.docx. [file ADVS-13-e22709-s001.docx]

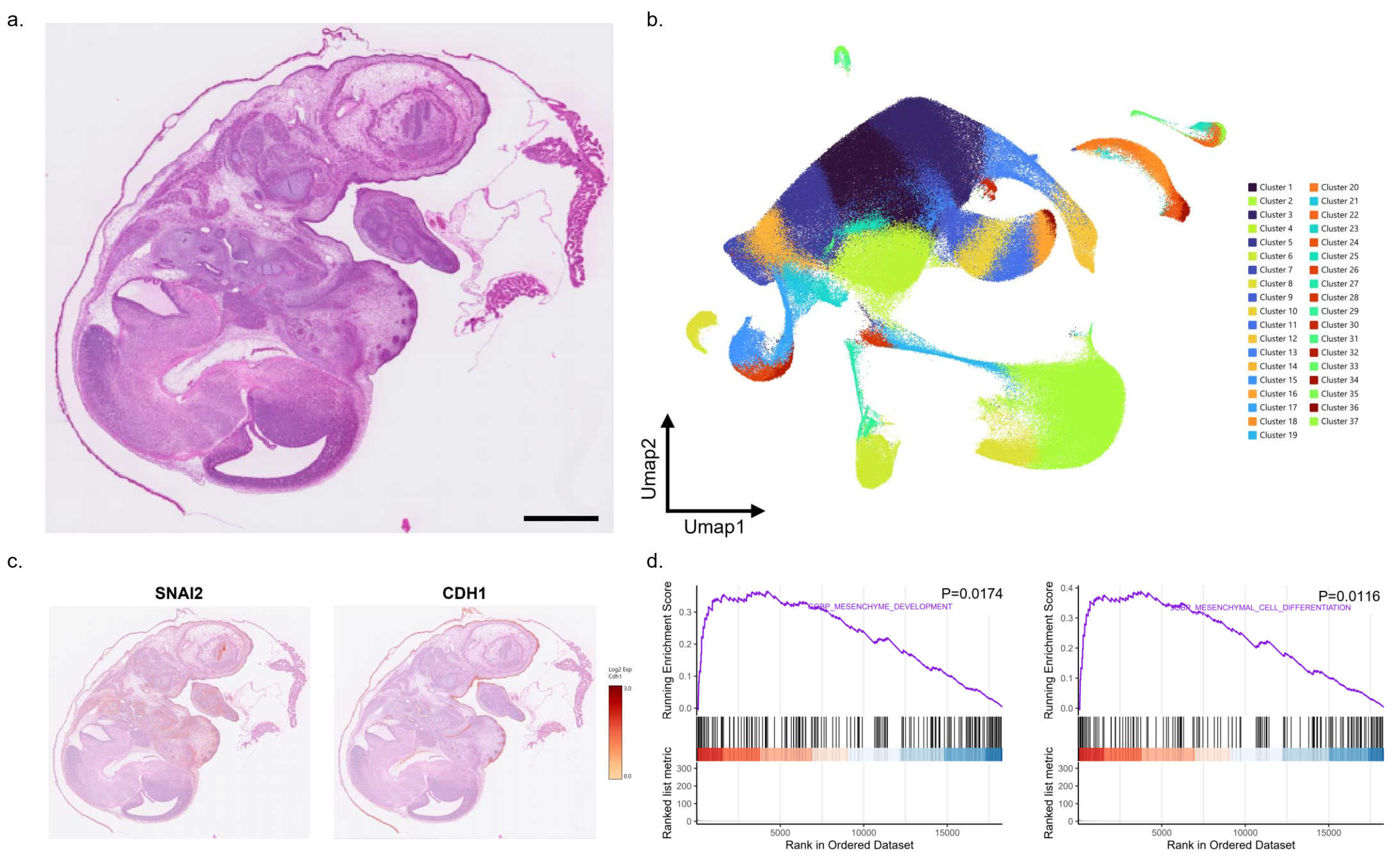


**Figure S1. Single cell spatial transcriptomic analysis of E15.5 mouse embryo**

a) H&E image of mouse embryo at E15.5 (bar= 1000 μm). b) E15.5 mouse embryo Umap projection of all subclusters. c) Spatial projection of SNAI2 and CDH1 gene expressions in E15.5 mouse embryo. d) GSEA analysis of DEGs between OAT+ intestinal epithelial cells and KRT5+ oral mucosal epithelial cells showing significant upregulation of TGF-β signaling pathway and EMT in intestinal epithelial cells.


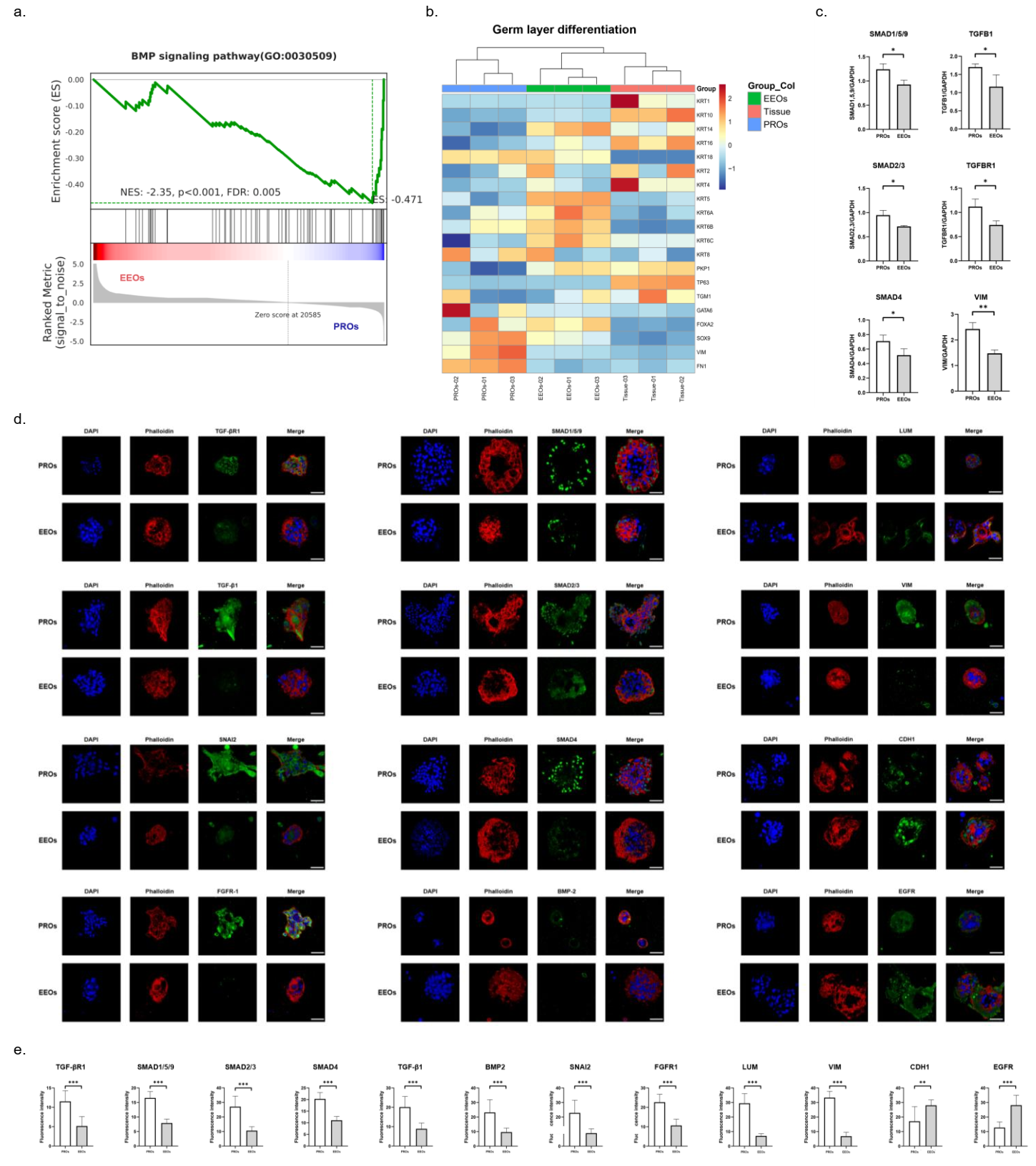


**Figure S2. TGF-β signaling pathway is significantly downregulated in EEOs, compared with PROs**

a) GSEA analysis of DEGs between EEOs and PROs showing significant downregulation of BMP and FGFR signaling pathway in EEOs. b) Western blotting quantitative analysis of expression levels of critical markers related to TGF-β signaling pathway between PROs and EEOs (data are shown as means ± SEM; t-test; n = 3). c) Clustering heatmap showing the expression pattern of critical genes related to germ layer differentiation between oral epithelial tissues, EEOs, and PROs. d) Representative ICC staining images of protein expression and localization of critical markers related to TGF-β signaling pathway, EMT, and ectodermal epithelial cell differentiation between EEOs and PROs (bar= 50 μm). e) ICC quantitative analysis of critical markers related to TGF-β signaling pathway, EMT, and ectodermal epithelial cell differentiation between EEOs and PROs (data are shown as means ± SEM; t-test; n = 3). *p <0.05, **p < 0.01, and ***p < 0.001. Statistical significance was set at p < 0.05.


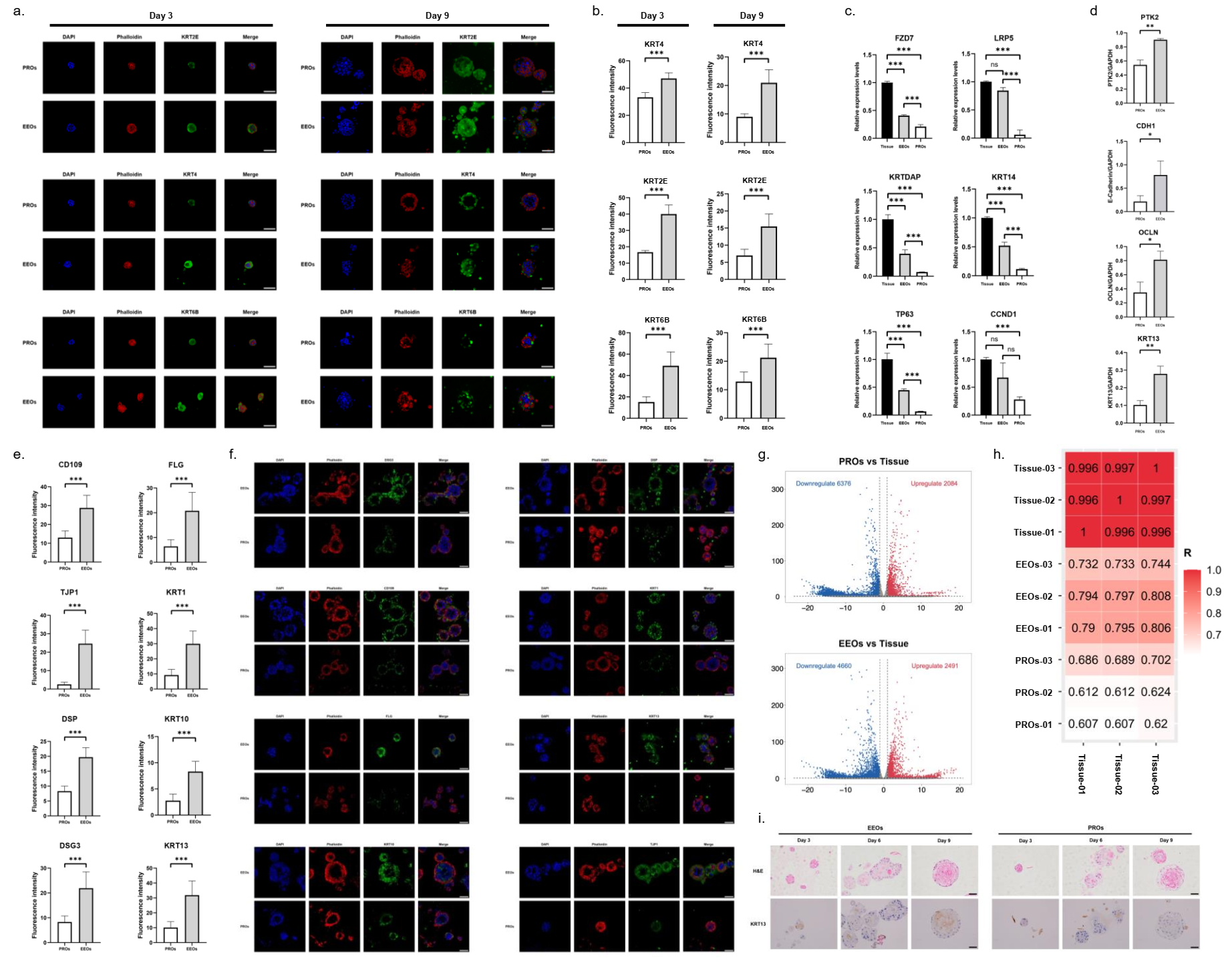


**Figure S3. EEOs shared greater similarity in gene and protein expression patterns with oral epithelial tissues, compared with PROs**

a) Representative ICC staining images comparing the protein expression and localization of KRT2E, KRT4, and KRT6B between EEOs and PROs on day 3 and 9 (bar= 100 μm). b) ICC quantitation of KRT2E, KRT4, and KRT6B between EEOs and PROs on day 3 and 9 (data are shown as means ± SEM; t-test; n = 3). c) qRT-PCR analysis of relative expression levels of critical genes related to epidermis development between oral epithelial tissues, EEOs, and PROs (data are shown as means ± SEM; t-test; n = 3). d) Western blotting quantitative analysis of expression levels of CDH1, PTK2, and KRT13 between EEOs and PROs (data are shown as means ± SEM; t-test; n = 3). e) ICC quantitation of DSG3, DSP, TJP1, FLG, CD109, KRT1, KRT10, and KRT13 between EEOs and PROs on day 12 of culturing (data are shown as means ± SEM; t-test; n = 3). f) Representative ICC staining images comparing the protein expression of DSG3, DSP, TJP1, FLG, CD109, KRT1, KRT10, and KRT13 between EEOs and PROs on day 12 (bar= 100 μm). g) Volcano plot showing the number of DEGs between oral epithelial tissues and EEOs, and between oral epithelial tissues and PROs respectively. h) Pearson correlation heatmap depicting the correlation of gene expression patterns between oral epithelial tissues, EEOs, and PROs. i) Representative H&E and IHC staining of epithelial tissues, EEOs, and PROs on days 3, 6 and 9, which comparing divergence in the expression of cornification marker KRT13 (bar= 50 μm). *p <0.05, **p < 0.01, and ***p < 0.001. Statistical significance was set at p < 0.05.


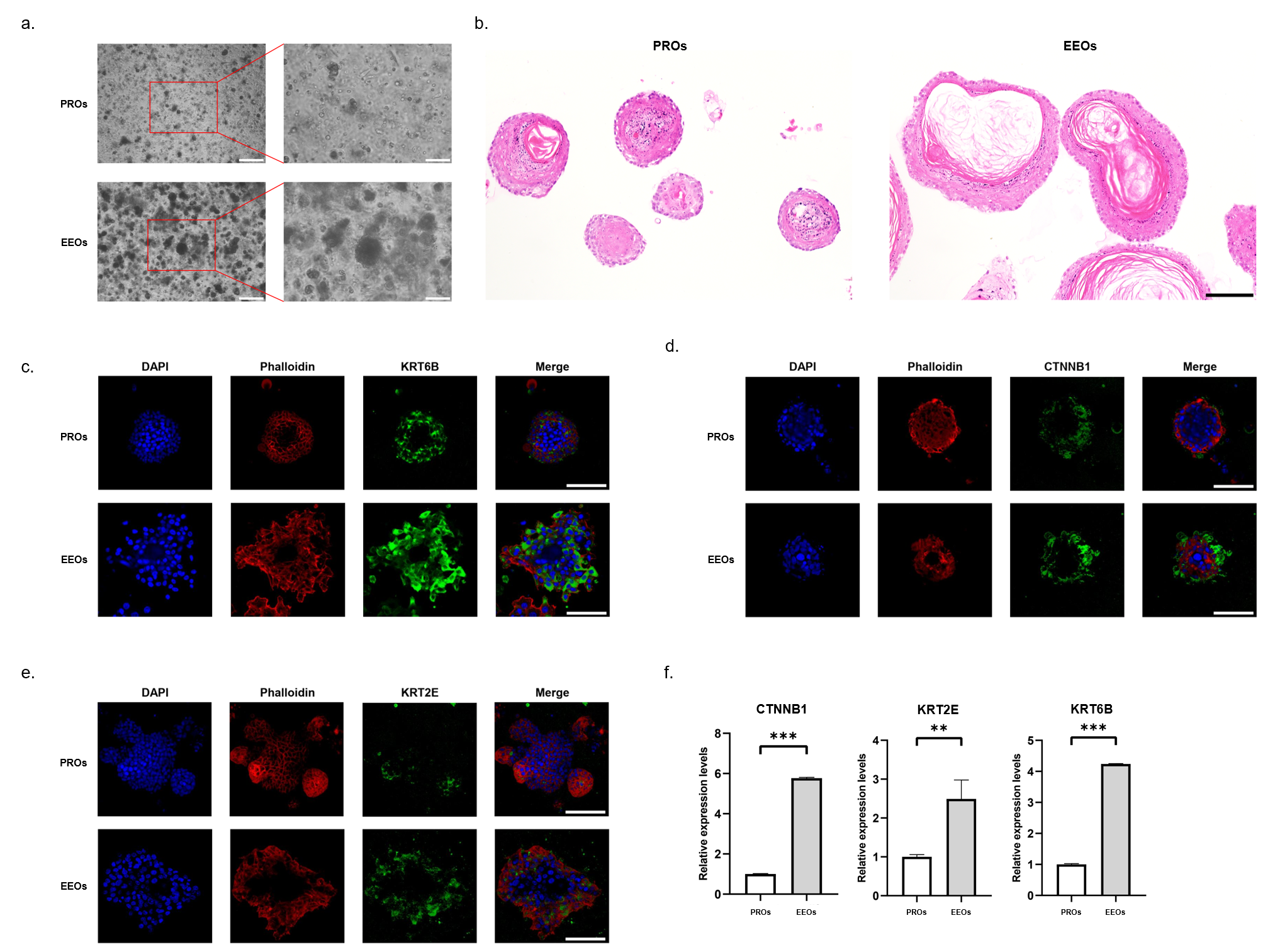


**Figure S4. Strategy for establishing EEOs can be extended to the establishment of epidermal organoids**

a) Representative light microscopy images of rat epidermal organoids fabricated by PRM and ectodermal-specific method (bar= 100 μm, 200 μm). b) Representative H&E staining of rat epidermal organoids fabricated by PRM and ectodermal-specific methods, depicting the formation of the stratum corneum (bar= 100 μm). c) Representative ICC staining images for detection of protein expression and localization of KRT6B of epidermal organoid culture (bar= 100 μm). d) Representative ICC staining images for detection of protein expression and localization of CTNNB1 of epidermal organoid culture (bar= 100 μm). e) Representative ICC staining images for detection of protein expression and localization of KRT2E of epidermal organoid culture (bar= 100 μm). f) ICC quantitation of CTNNB1, KRT2E, and KRT6B of epidermal organoids fabricated by PRM and ectodermal-specific method (data are shown as means ± SEM; t-test; n = 3). *p <0.05, **p < 0.01, and ***p < 0.001. Statistical significance was set at p < 0.05.


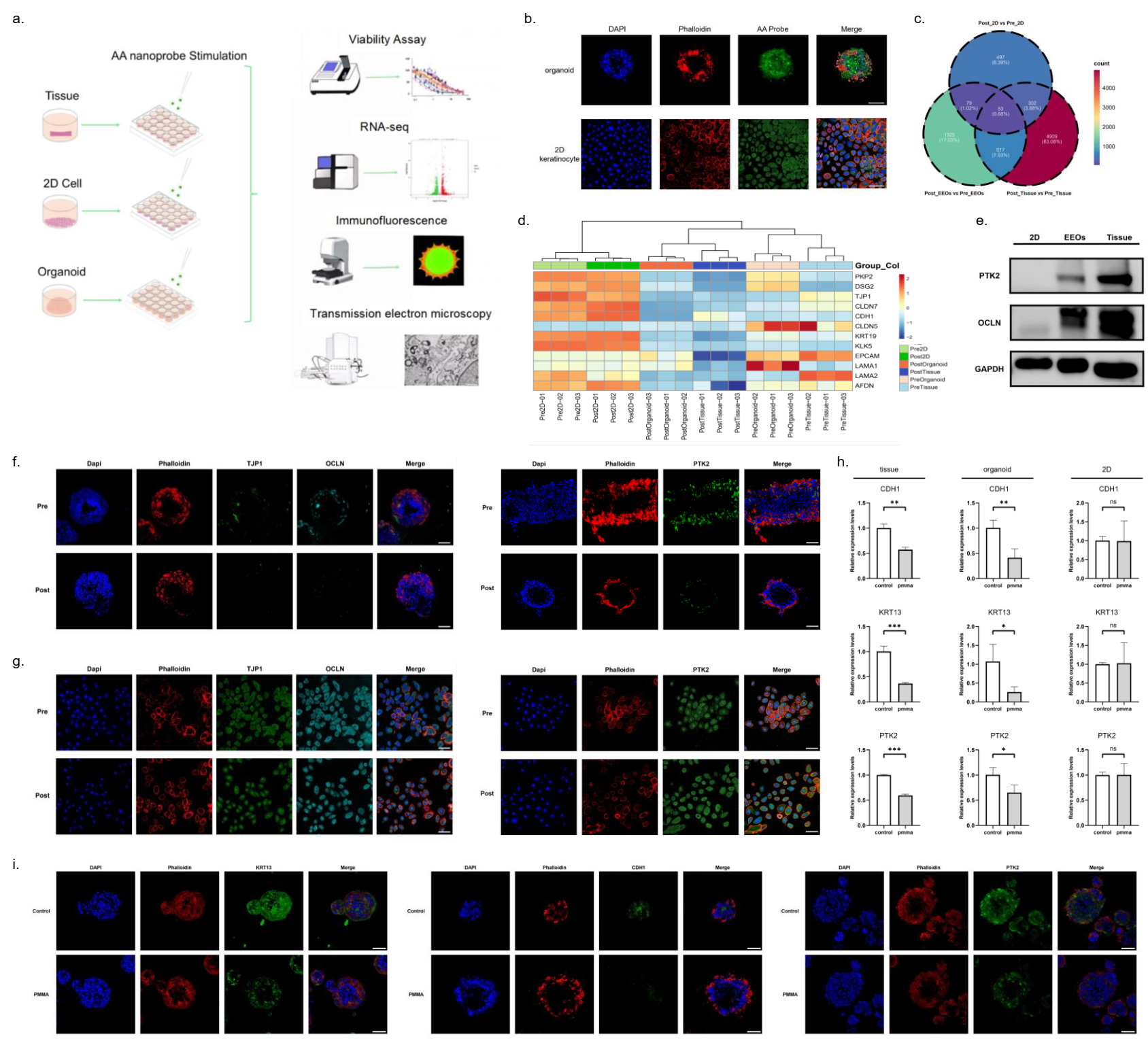


**Figure S5. Comparing the sensitivity of nanomaterials toxicological screening between EEOs and 2S cultured keratinocytes**

a) Schematic diagram depicting the procedure for nanoprobe toxicity screening. b) Representative images of AA fluorescent probes penetrating EEOs and 2D cultured keratinocytes (bar= 50 μm). c) Venn plot depicting the count of DEGs between oral epithelial tissues, EEOs, and 2D cultured keratinocytes before and after exposure to AA nanoprobes. d) Clustering heatmap depicting alterations in the expression levels of critical genes related to cellular junctions between oral epithelial tissues, EEOs, and 2D cultured keratinocytes before and after exposure to AA nanoprobes. e) Representative western blotting images comparing expression levels of critical proteins related to epithelial cell junctions between oral epithelial tissues, EEOs, and 2D cultured keratinocytes. f) Representative ICC staining images comparing the expression of cellular junction proteins TJP1, OCLN, and PTK2 in EEOs before and after exposure to AA nanoprobes (bar= 100 μm). g) Representative ICC staining images comparing the expression of cellular junction proteins TJP1, OCLN, and PTK2 in 2D cultured keratinocytes before and after exposure to AA nanoprobes (bar= 100 μm). h) qRT-PCR analysis of the relative expression levels of CDH1, KRT13, and PTK2 between oral epithelial tissues, EEOs, and 2D cultured keratinocytes before and after exposure to PMMA nanoparticles (data are shown as means ± SEM; t-test; n = 3). i, Representative ICC staining images comparing the expression of barrier function markers KRT13, CDH1, and PTK2 in EEOs before and after exposure to PMMA nanoparticles (bar= 100 μm). *p <0.05, **p < 0.01, and ***p < 0.001. Statistical significance was set at p < 0.05.


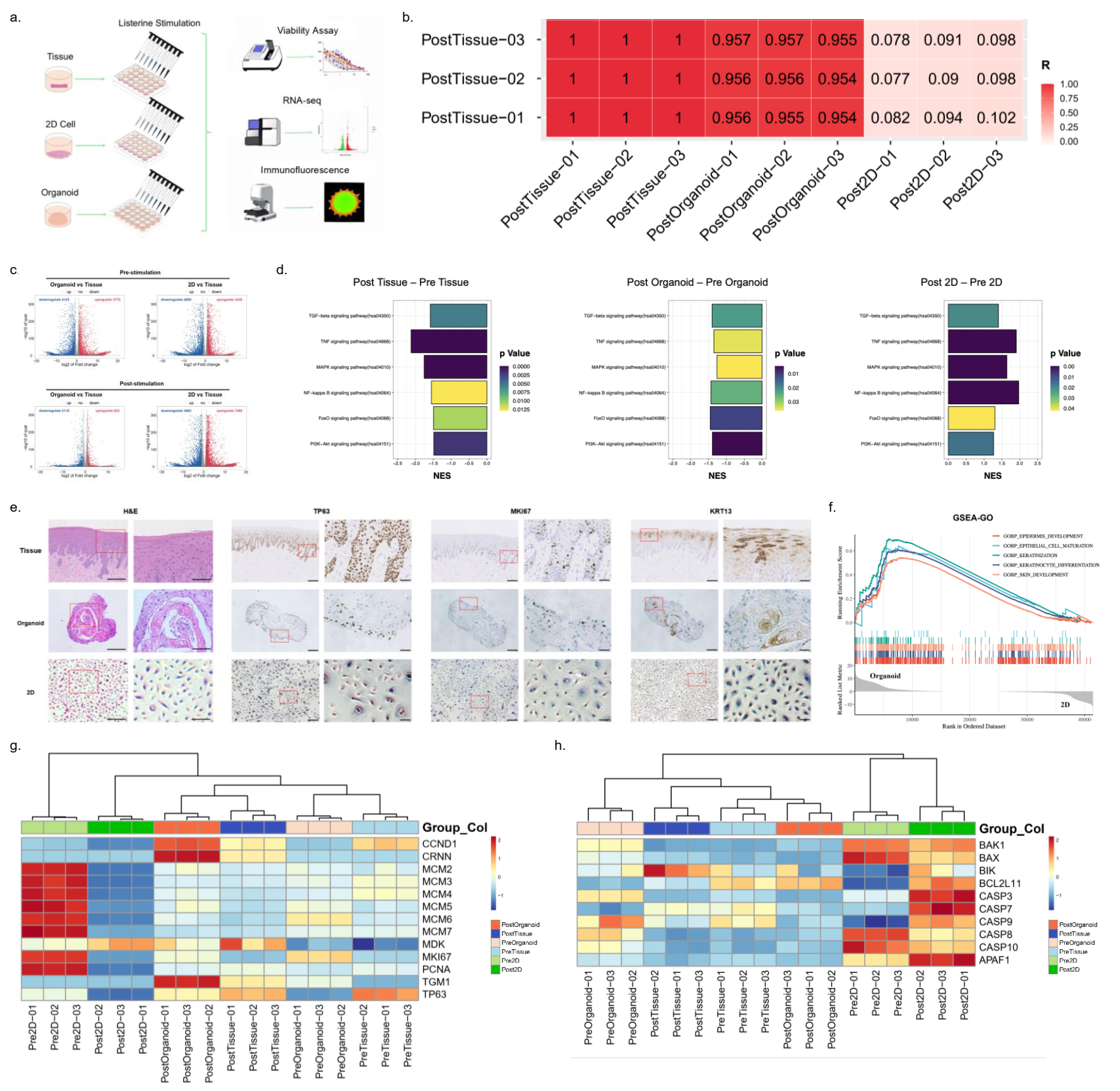


**Supplementary figure 6. Comparing the accuracy of pharmaceutical toxicological screening between EEOs and 2D cultured keratinocytes**

a) Schematic diagram depicting the procedure for pharmaceutical toxicological screening. b) Pearson correlation heatmap depicting the correlation of gene expression patterns between oral epithelial tissues, EEOs, and 2D cultured keratinocytes posterior to Listerine® stimulation. c) Volcano plot depicting the number of DEGs between EEOs and oral epithelial tissues, and between 2D cultured keratinocytes and oral epithelial tissues prior and posterior to Listerine® stimulation. d) GSEA-KEGG analysis of DEGs between post-stimulated and pre-stimulated oral epithelial tissues, EEOs, and 2D cultured keratinocytes. This revealed significant downregulation of TGF-β, TNF, MAPK, PI3K-AKT, NF-κB, and FOXO signaling pathways in oral epithelial tissues and EEOs after Listerine® stimulation, but significant upregulation of TGF-β, TNF, MAPK, PI3K-AKT, NF-κB, and FOXO signaling pathways in 2D cultured keratinocytes after Listerine® stimulation. e) Representative H&E and IHC staining of oral epithelial tissues, EEOs and 2D cultured keratinocytes (bar= 200 μm, 40 μm). f) GSEA-GO analysis of DEGs between EEOs and 2D cultured keratinocytes showing significant upregulation of biological functions including epidermis development, epithelial cell maturation, keratinization, keratinocyte differentiation, and skin development in EEOs. g) Clustering heatmap depicting the alteration of apoptosis-related gene expression patterns between oral epithelial tissues, EEOs. and 2D cultured keratinocytes after Listerine® stimulation. h) Clustering heatmap depicting the alteration of proliferation-related gene expression patterns between oral epithelial tissues, EEOs, and 2D cultured keratinocytes after Listerine® stimulation.


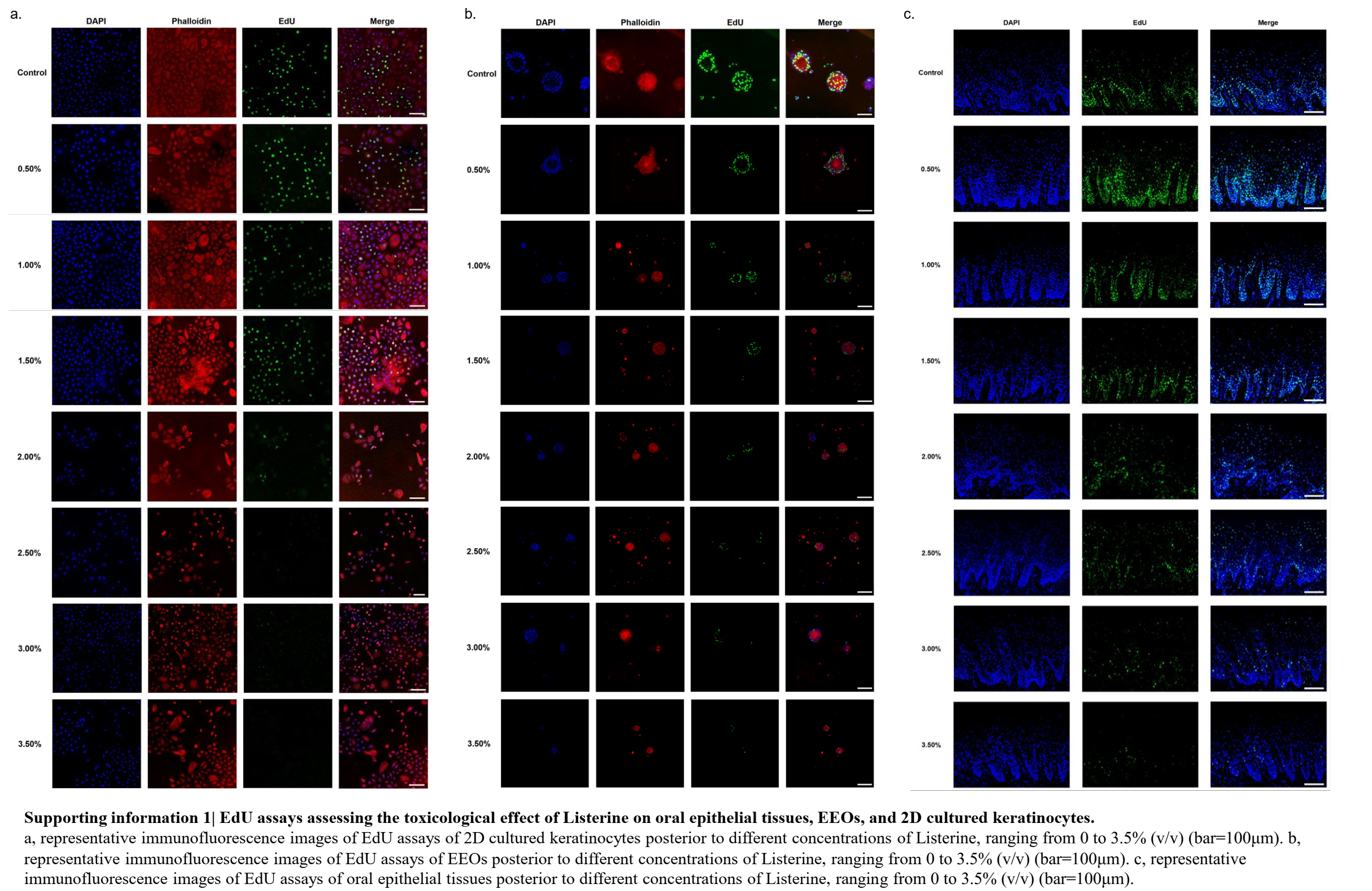


**Supporting information 1. EdU assays assessing the toxicological effect of Listerine® stimulation on oral epithelial tissues, EEOs, and 2D cultured keratinocytes**

a) Representative immunofluorescence images of EdU assays of 2D cultured keratinocytes posterior to different concentrations of Listerine® stimulation, ranging from 0 to 3.5% (v/v) (bar= 100 μm). b) Representative immunofluorescence images of EdU assays of EEOs posterior to different concentrations of Listerine® stimulation, ranging from 0 to 3.5% (v/v) (bar= 100 μm). c) Representative immunofluorescence images of EdU assays of oral epithelial tissues posterior to different concentrations of Listerine® stimulation, ranging from 0 to 3.5% (v/v) (bar= 100 μm).


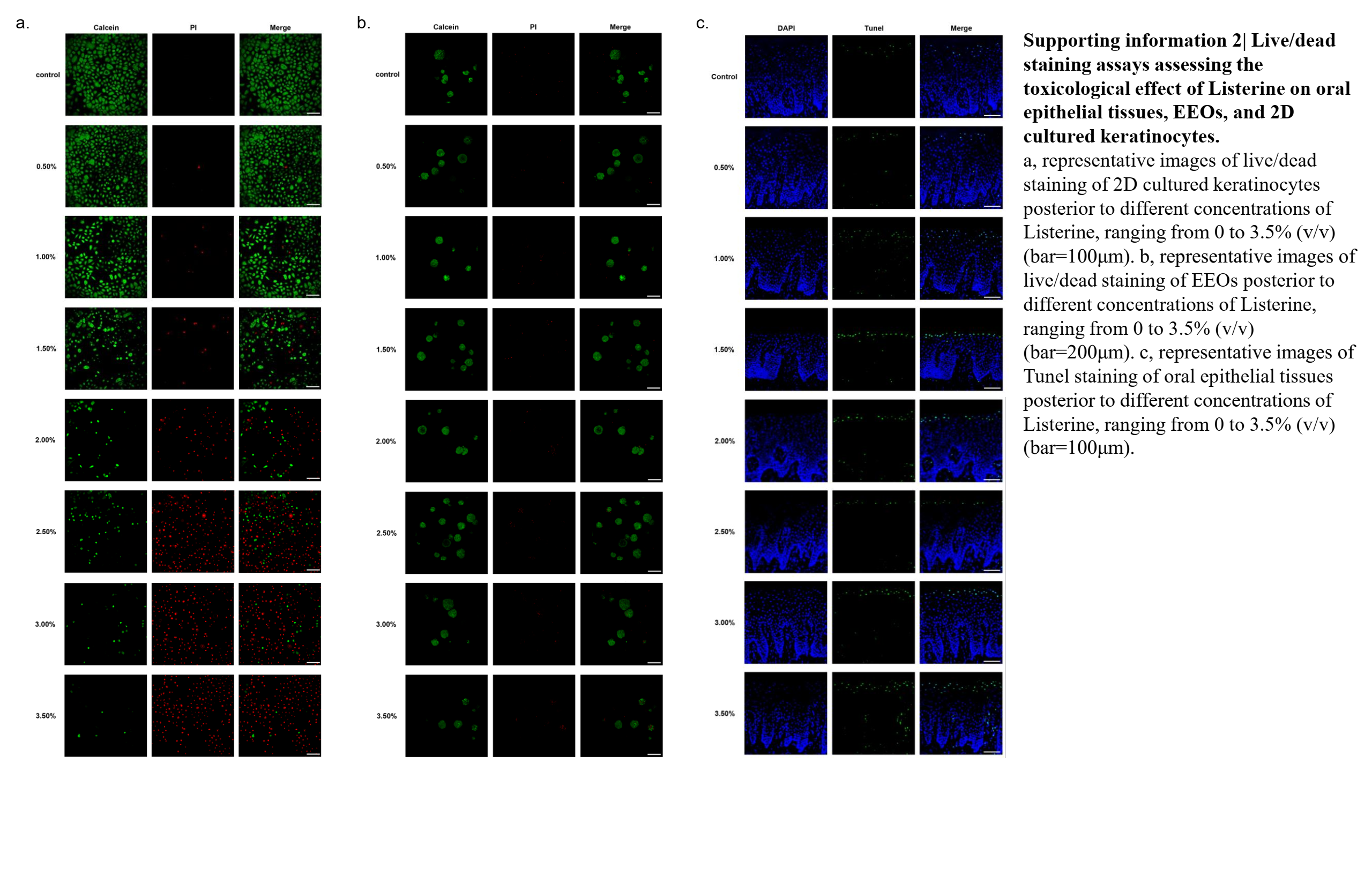


**Supporting information 2. Live/dead staining assays assessing the toxicological effect of Listerine® stimulation on oral epithelial tissues, EEOs, and 2D cultured keratinocytes**

a) Representative immunofluorescence images of live/dead staining of 2D cultured keratinocytes posterior to different concentrations of Listerine® stimulation, ranging from 0 to 3.5% (v/v) (bar= 100 μm). b) Representative immunofluorescence images of live/dead staining of EEOs posterior to different concentrations of Listerine® stimulation, ranging from 0 to 3.5% (v/v) (bar= 200 μm). c) Representative immunofluorescence images of Tunel staining of oral epithelial tissues posterior to different concentrations of Listerine® stimulation, ranging from 0 to 3.5% (v/v) (bar= 100 μm).


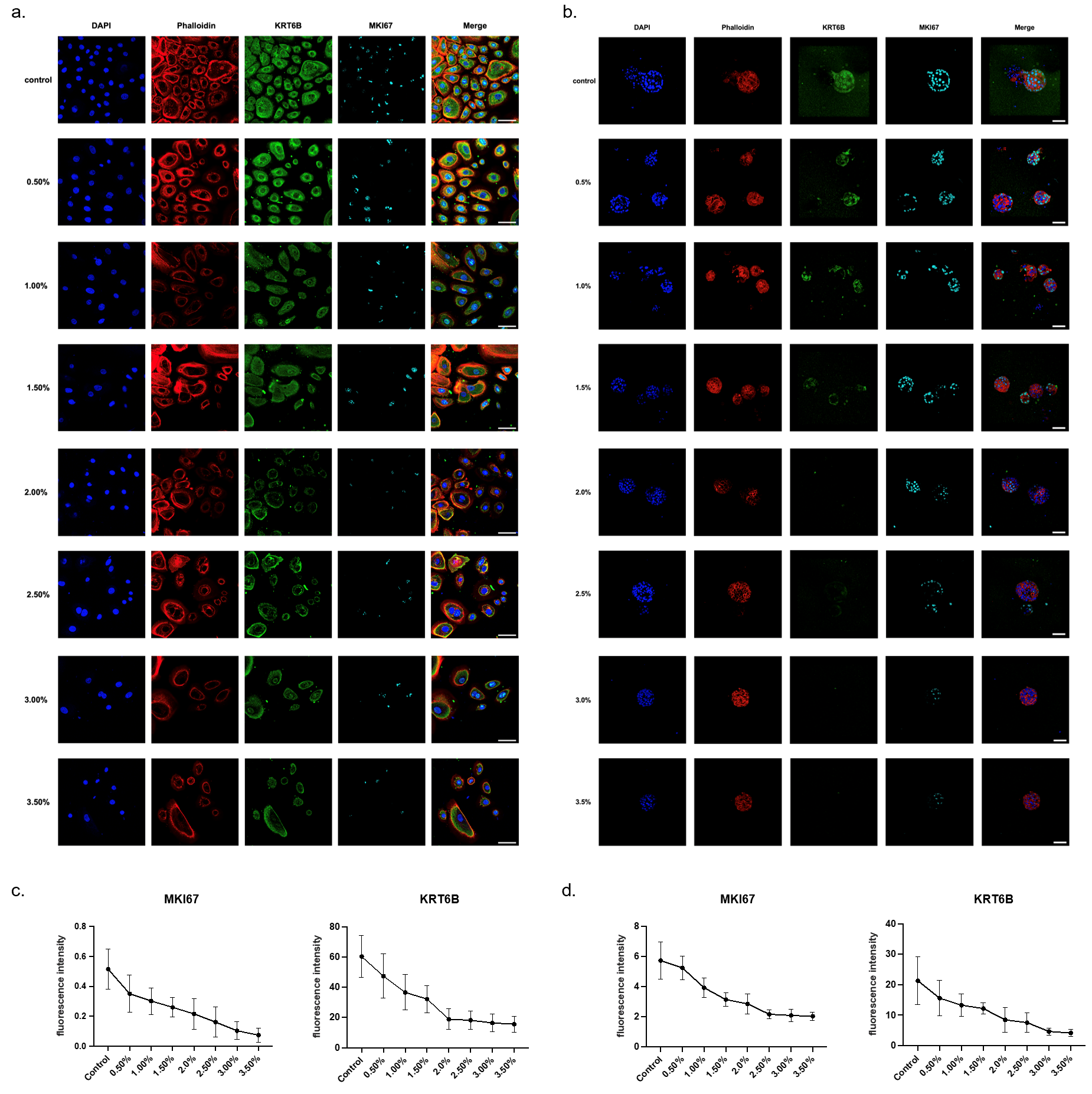


**Supporting information 3. ICC assays of MKI67 and KRT6B expression levels in oral epithelial tissues, EEOs and 2D cultured keratinocytes prior and posterior to Listerine® stimulation**

a) Representative ICC staining images of 2D cultured keratinocytes posterior to different concentrations of Listerine® stimulation, ranging from 0 to 3.5% (v/v) (bar= 50μm). b) Representative ICC staining images of EEOs posterior to different concentrations of Listerine® stimulation, ranging from 0 to 3.5% (v/v) (bar= 50 μm). c) ICC quantitation assays of MKI67 and KRT6B of 2D cultured keratinocytes posterior to different concentrations of Listerine® stimulation, ranging from 0 to 3.5% (v/v). d) ICC quantitation assays of MKI67 and KRT6B of EEOs posterior to different concentrations of Listerine® stimulation, ranging from 0 to 3.5% (v/v).


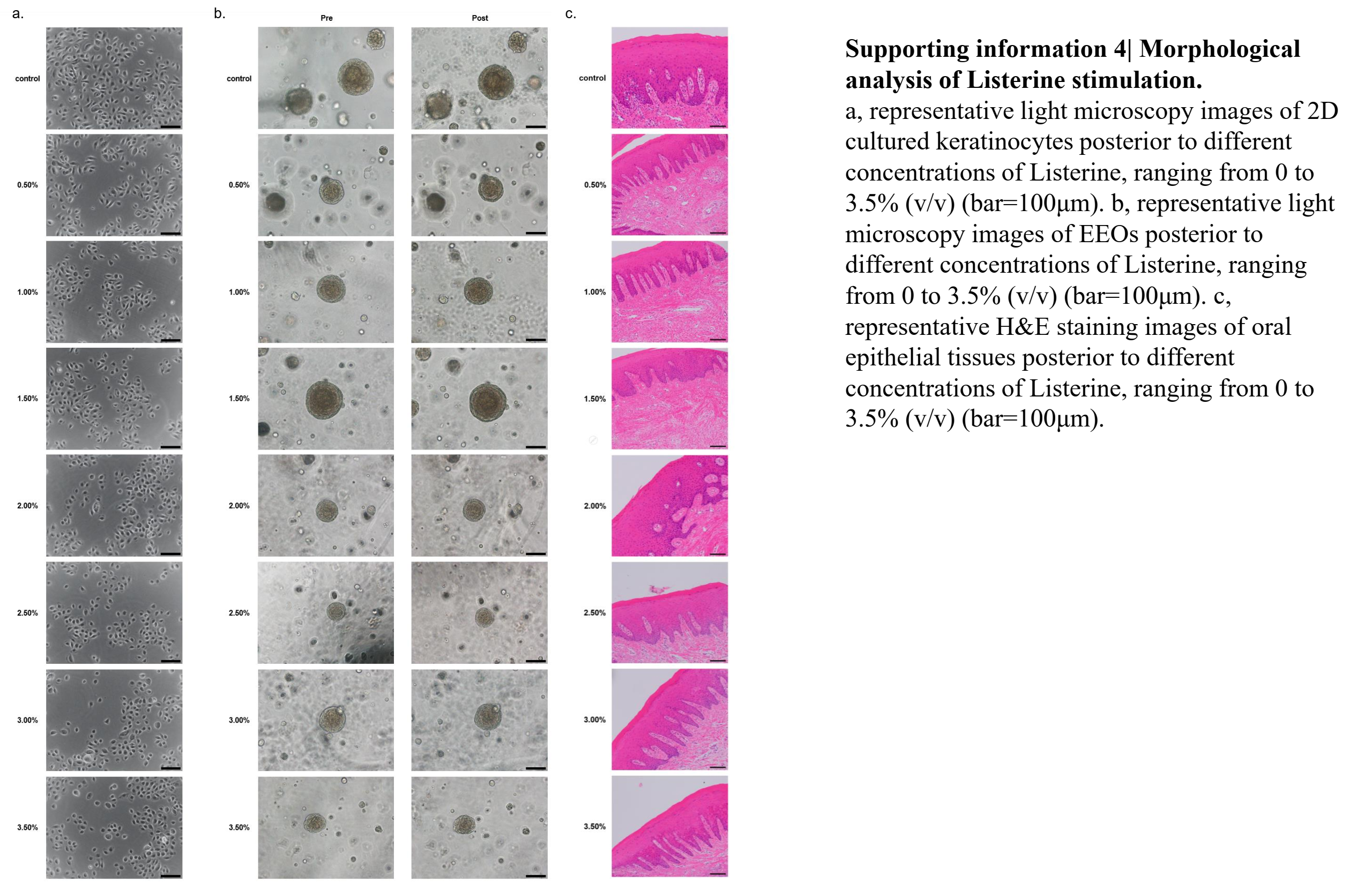


**Supporting information 4. Morphological analysis of oral epithelial tissues, EEOs and 2D cultured keratinocytes prior and posterior to Listerine® stimulation**

a) Representative light microscopy images of 2D cultured keratinocytes posterior to different concentrations of Listerine® stimulation, ranging from 0 to 3.5% (v/v) (bar= 100 μm). b) Representative light microscopy images of EEOs posterior to different concentrations of Listerine® stimulation, ranging from 0 to 3.5% (v/v) (bar= 100 μm). c) Representative H&E staining images of oral epithelial tissues posterior to different concentrations of Listerine® stimulation, ranging from 0 to 3.5% (v/v) (bar= 100 μm).

**Materials and methods**

**EXPERIMENTAL MODEL AND SUBJECT DETAILS**

**Human oral mucosal epithelium**

Human oral mucosal epithelia were obtained from implant surgeries and preserved at 4℃ in advanced DMEM/F12 medium (Gibco 12634-010) supplemented with 5% penicillin/streptomycin solution (Gibco: 15140122) and 10μmol/L Y-27632 (MCE HY10071) for further assays. Gingival tissue collection was approved by the Human Ethics Committee of Peking University School and Hospital of Stomatology (Approval number: PKUSSIRB-202282166). The donors who participated in our study volunteered and signed informed consent forms.

**Rat dorsal epithelium**

Dorsal epithelia were extracted from 8-week-old male Sprague-Dawley (SD) rats (Charles Rivers). Rats were domesticated in SPF laboratory so as to ensure they were healthy and free of infectious agents. Animals had free access to food and water during the study. SD rats were euthanized by asphyxiation with CO2, and dorsal epithelia were surgically extracted and preserved at 4℃ in advanced DMEM/F12 medium (Gibco 12634-010) supplemented with 5% penicillin/streptomycin solution (Gibco: 15140122) and 10μmol/L Y-27632 (MCE HY10071) for further assays. All animal experiments were approved by the Institutional Animal Care and Use Committee of the Peking University (Approval number: LA2022394). All efforts were made to minimize the suffering of animals.

**METHOD DETAILS**

**Epithelial cell isolation and organoid culture**

Human oral mucosal epithelia and rat dorsal epithelia were obtained and preserved following the protocol described above. Briefly, epithelial tissues were washed with ice-cold PBS supplemented with 5% penicillin/streptomycin solution for 3 times. Tissues were divided into 1mm^2^ pieces, and the lamina propria was removed by applying Dispase II (2.5 mg/ml, Roche: 04942078001). Subsequently, tissues were digested with a combination of 1 mg/ml hyaluronidase (Sigma H3506) and 2 mg/ml collagenase I (Sigma SCR103) for 2 hours. Digestion was terminated by adding DMEM/F12 containing 5% fetal bovine serum (Procell 164210-50). The suspension was strained over a 100 μm filter and centrifuged at 200 g for 5 min. The sediment was resuspended in Matrigel (Corning #354234) and droplets of 50μL were embedded in 24-well plates. The plates were inverted and placed in 37℃ incubators for 30 min to facilitate the solidifying of matrigel. The gels were then overlayed with 500μL of culture medium. The composition of conventional organoid culture medium and ectodermal specific culture medium are listed in Supplementary Table 1 and 2. 10 µmol/L Rho-associated kinase (ROCK) inhibitor Y-27632 was added to the medium for the first 7 days of culturing so as to aid the outgrowth of organoids^15,16^. Organoids reached a mature state on day 12 of culturing, and were harvested for further experiments (e.g., RNA-sequencing, IHC/ICC assays, real time PCR, Western blot assays) or passaged. The tissue origin and culture conditions of previously reported organoids (PROs) and ectodermal epithelial organoids (EEOs) are identical in our study so as to exclude other interfering factors.


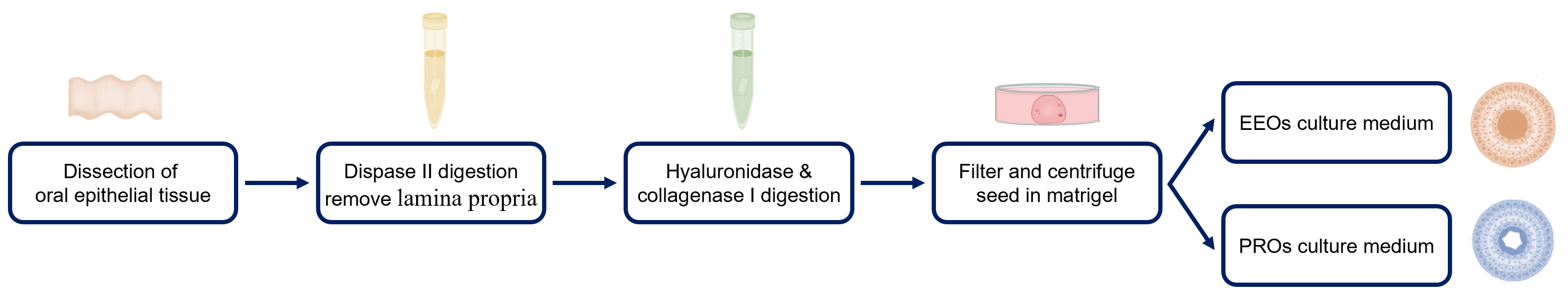


Supplementary material figure 1| organoid culture workflow

For organoid passaging, the matrigel droplet was disrupted with a pipette, organoids were further enzymatically dissociated with TrypLE Express (Gibco #12605-010) at 37℃ for 30 min. Digestion was terminated by adding DMEM/F12 containing 5% fetal bovine serum. The suspension was strained over a 100 μm filter and centrifuged at 200 g for 5 min. Passaging was completed by resuspending the sediment with Matrigel and embedding the droplets into culture plates.

**Monolayer keratinocyte culture**

Epithelial tissues obtained from human oral mucosa were digested following the same protocols mentioned above. The sediments were resuspended in oral keratinocyte medium (Science Cell: 2611) and cultured in 6-well plates. Primary keratinocytes took 12-14 days to reach 80% confluence, passaged keratinocytes took 3 days to reach 80% confluence. Subsequent examinations were carried out on P3 keratinocytes when the cells reached 80% confluence. For keratinocyte passaging, 0.25% Trypsin (Gibco #25200-072) was added to the culture plate when cells reached 80% confluency. Digestion was terminated by adding DMEM/F12 containing 5% fetal bovine serum. The suspension was strained over a 100 μm filter and centrifuged at 200 g for 5 min. Passaging was completed by resuspending the sediment with oral keratinocyte medium and placing into 6-well plates.

**RNA collection**

Prior to RNA collection of organoids, the matrigel droplet was disrupted with a pipette, suspension was washed with ice-cold PBS for 3 times and centrifuged at 200 g for 5 min. Total RNA was extracted from the sediment using TRIzol Reagent (Invitrogen #15596026) according to the manufacturer’s instructions. For RNA collection of monolayer cultured keratinocytes, cells were washed with ice-cold PBS for 3 times and TRIzol Reagent was added into the culture plates. The quality and quantity of the RNA samples obtained were analyzed using a bio-photometer (Thermo Fisher Scientific™ NanoDrop8000). RNA samples were reserved at -80℃ for sequencing or qRT-PCR assays.

**RNA sequencing**

Posterior to total RNA extraction, Poly (A) RNA was purified from 1 μg total RNA per sample using Dynabeads Oligo (dT) (Thermo Fisher #25-61005) for the final cDNA library with average insert size 300 ± 50 bp. Purified RNA was subjected to the 2× 150 bp paired-end sequencing (PE150) on an Illumina Novaseq™ 6000 following the recommended protocol (LC-Bio Technology CO., Ltd., Hangzhou, China). Sequence quality was verified using Fastp software (<https://github.com/OpenGene/fastp>). The reads were mapped to the reference genome of Homo sapiens GRCh38 using HISAT2 (<https://daehwankimlab.github.io/hisat2/>). The mapped reads of each sample were assembled using StringTie, and the comprehensive transcriptome was reconstructed using Gffcompare. The FPKM of expression level for mRNAs was calculated using StringTie. Significantly differential expressions were defined as |Log2 fold change| > 1 and p-value < 0.05 (parametric F-test) by R package DESeq2. Gene set enrichment analysis (GSEA) of significantly differently expressed genes were performed with the R package clusterprofiler. Principal component analysis (PCA) and correlation analysis were performed with the R package FactoMineR and factoextra. The heatmaps of significantly differently expressed genes were drawn with the R package ggplot2.

**Spatial transcriptomics analysis of E15.5 mouse embryo**

Visium HD spatial transcriptomics data was downloaded from 10x Genomics (<https://www.10xgenomics.com/cn/datasets/visium-hd-cytassist-gene-expression-libraries-of-mouse-embryo>). Intestinal and oral mucosal epithelial cell subclusters were identified by differential enrichment analysis and marker gene projection. Spatial projection of marker genes was carried out with Loupe Browser Version 9.0 (<https://www.10xgenomics.com/support/cn/software/loupe-browser/latest>). Differential enrichment analysis was carried out by R package DESeq2. Gene set enrichment analysis (GSEA) of significantly differently expressed genes were performed with the R package clusterprofiler.

**cDNA Synthesis and Quantitative real-time PCR**

Following total RNA extraction, RNA samples were reverse transcribed into complementary DNA (cDNA) using a Reverse Transcription kit (Takara Bio #RR037A). Quantitative real-time polymerase chain reaction (qRT-PCR) was carried out using a FastStart Universal SYBR Green Master Mix (Rox) system with QuantStudio Design & Analysis Desktop Software (Thermo Fisher Scientific). Primers were designed according to the published sequences and listed in Supplementary Table 3. Data were normalized based on the Glyceraldehyde-3-phosphate dehydrogenase (GAPDH) expression levels.

**Western blot assays**

The total protein of epithelial organoids or monolayer cultured keratinocytes were extracted with RIPA lysis buffer (Beyotime #P0039) with a protease inhibitor cocktail (PMSF Solarbio #P0100) on ice. The protein concentration of each sample was quantified using Pierce™ Dilution-Free™ Rapid Gold BCA protein assay kit (Thermo Fisher Scientific #A55860). Protein samples were supplemented with six times SDS loading buffer (Beyotime #P0015F) and heated at 100 °C for 5 min. The total protein extract was subject to 10% (w/v) sodium dodecyl sulfate polyacrylamide gel electrophoresis with 40 μg in each lane. Afterwards, the separated proteins were transferred to PVDF membranes and blocked with 5% (w/v) skimmed milk. The membranes were incubated in primary antibodies (diluted with 5% skimmed milk) at 4 °C for 12 h and then rinsed with Trisbuffered saline/Tween 20 (TBST). Then, the membranes were incubated with horseradish peroxidase (HRP) conjugated secondary antibodies (Beyotime #A0208, #A0216) for 1 h at RT and rinsed with TBST. Details of primary and secondary antibodies are listed in Supplementary Table 4. The immunoreactive protein bands were visualized with an eECL Super-sensitive chemiluminescent substrate (Beyotime #P0018AS) on a chemiluminescence immunoassay analyzer (Bio-Rad #Chemidoc MP). The relative expression levels of target proteins were normalized to blotted house-keeping protein Gapdh. The gray scale intensity of blotted bind was analyzed and obtained using software ImageJ.

**Immunocytochemistry assays**

Epithelial organoids and monolayer cultured keratinocytes were rinsed with ice-cold PBS, fixed in 4% (w/v) paraformaldehyde for 20 min at room temperature (RT), and rinsed with ice-cold PBS again. Then, samples were permeabilized with 0.1% (w/v) Triton X-100 (diluted with PBS) for 10 min and blocked with 3% (w/v) bovine serum albumin (BSA; diluted with PBS) for 1 h at RT. Subsequently, samples were incubated in primary antibodies diluted with 3% (w/v) BSA solution for 12 h at 4°C. After careful removal of primary antibodies by rinsing with ice-cold PBS, samples were incubated in secondary antibodies (Abcam #ab150115, #ab150113, 1:200, diluted with PBS) along with DAPI (Solarbio #C0060, 1:100, diluted with PBS) and Phalloidin (Solarbio #CA1610, 1:200, diluted with PBS) for 1 h at RT. Details of primary and secondary antibodies are listed in Supplementary Table 4. Following extensive rinsing with PBS, samples were imaged with confocal microscopy (Leica SP8-STED). The quantifications of immunofluorescence were analyzed with LAS X Software (Media Cybernetics).

**Barrier permeability assays**

Epithelial barrier permeability assays were carried out on EEOs and PROs. 100µg/mL isothiocyanate fluorescein-dextran (Sigma-Aldrich #FD4-FITC / FD10S-FITC) was added to the culture medium. Organoids were incubated at 37℃ for 1h, and washed for 3 times with PBS. The barrier permeability of EEOs and PROs were assessed by confocal microscopy (Leica SP8-STED). The quantifications of immunofluorescence were analyzed with LAS X Software (Media Cybernetics).

**Trans-epithelium electric resistance (TEER) assays**

Trans-epithelium electric resistance (TEER) assays were carried out on EEOs and PROs. Organoids were harvested on day 12 of culturing, and enzymatically dissociated with TrypLE Express (Gibco #12605-010) at 37℃ for 30 min. Digestion was terminated by adding DMEM/F12 containing 5% fetal bovine serum. The suspension was strained over a 100 μm filter and centrifuged at 200 g for 5 min. The sediment was resuspended in 20% matrigel and seeded in the upper chamber of 24-well Transwell plates. Resistance was monitored on day 3, day 6, day 9, and day12 of culturing with Millicell ERS-2 system (Merck MERS00002). The unit area resistance was calculated by Resistance (Ω) x 0.3 (cm^2^).

**Epithelial barrier stimulation assays of Listerine®**

Epithelial barrier stimulation assays of Listerine® were carried out on oral mucosa epithelial tissues, ectodermal epithelial organoids, and 2D cultured keratinocytes. With regards to oral mucosa epithelial tissues, tissues were incubated in DMEM/F12 supplemented with different concentrations of Listerine® ranging from 0% to 3.5% (v/v). With regard to ectodermal epithelial organoids, and monolayer cultured keratinocytes, samples were passaged to P3 and incubated in 24-well plates. Culture medium supplemented with different concentrations of Listerine® ranging from 0% to 3.5% (v/v) were added. All samples were incubated at 37℃ for 4 h, and further subject to viability assays. The toxicological effect of Listerine® on epithelial tissues were assessed by EdU quantitation. The toxicological effect of Listerine® on epithelial organoids were assessed by ATP synthesis quantitation. The toxicological effect of Listerine® on monolayer cultured keratinocytes were assessed by CCK-8 assays. EEOs, 2D models, and human biopsies were derived from identical patients and tested under the same chemical exposure so as to exclude other interfering factors.

**Epithelial barrier stimulation tests of nanomaterials**

Epithelial barrier stimulation tests of nanoparticles were carried out on oral mucosa epithelial tissues, ectodermal epithelial organoids, conventional epithelial organoids, and 2D cultured keratinocytes. Oral mucosa epithelial tissues were incubated in DMEM/F12 culture medium supplemented with 2mg/mL (AA) neutral red fluorescent nanoprobes / 2mg/mL PMMA nanoparticles at 37℃ for 4 h. Organoids and 2D cultured keratinocytes, samples were passaged to P3 and incubated in culture medium supplemented with 2mg/mL acrylic acid (AA) neutral red fluorescent nanoprobes / 2mg/mL PMMA nanoparticles at 37℃ for 4 h. The penetration of nanomaterials into the epithelial barrier was assessed by immunofluorescence assays. The effects of nanomaterials on epithelial cellular junctions were assessed by immunohistochemistry assays, immunofluorescence assays, and RNA-sequencing. EEOs, 2D models, and human biopsies were derived from identical patients and tested under the same chemical exposure so as to exclude other interfering factors.

**EdU cell proliferation assays and quantification**

EdU assays were carried out to assess the growth rate of organoids as well as the toxicity effects of Listerine® on organoids, monolayer cultured keratinocytes and epithelial tissues. Samples were incubated with EdU (1:1000, diluted with culture medium) for 2 hours, rinsed with ice-cold PBS and fixed in 4% (w/v) paraformaldehyde or 20 min at RT. Then, samples were rinsed with glycine, permeabilized with 0.1% (w/v) Triton X-100 for 10 min, and blocked with 3% (w/v) BSA for 1 h at RT. Subsequently, samples were stained with FITC-labeled Apollo reaction solution (RiboBio #C10310-3) for 30 min at RT. Prior to imaging, samples were rinsed with ice-cold PBS and stained with Hoechst33342 for nucleus staining. With regards to epithelial tissues, samples were fixed, embedded, and sectioned prior to staining. Samples were imaged with confocal microscopy (Leica SP8-STED), and the staining of EdU was quantified by the ratio of cells positive with EdU staining.

**ATP synthesis quantitation**

ATP levels were measured so as to assess the growth rate of organoids as well as the toxicity effects of Listerine® on organoids. CellTiter-Glo 3-D Reagent (Promega, catalog no. G9681) was added into organoid culture medium. The culture medium was then sheared using a pipette for 5 min and placed at 25℃ for samples to dissociate. Luminescence was measured using a Spark multimode microplate reader according to the manufacturer's instructions.

**CCK-8 assays**

CCK-8 assays were carried out to assess the toxicity effects of Listerine® on monolayer cultured keratinocytes. CCK-8 reagent (Beyotime #C0037) was added to the culture medium of monolayer cultured keratinocytes and incubated at 37℃ for 1 h. The absorbance of the solution at 450 nm was measured using a microplate reader according to the manufacturer's instructions.

**CCK-3D assays**

CCK-3D assays were carried out to assess the growth rate of organoids. CCK-3D reagent (Beyotime #C0049S) was added to the culture medium of organoids and incubated at 37℃ for 1 h. The absorbance of the solution at 450 nm was measured using a microplate reader according to the manufacturer's instructions.

**Live/Dead assays**

Live/Dead assays were carried out to assess the toxicity effects of Listerine® on organoids and monolayer cultured keratinocytes. An equivalent volume of Cyto3D reagent (TheWell BIOSCIENCE #BM01) was added to the culture medium of organoids, and incubated at 37℃ for 10 min. An equivalent volume of Calcein/PI reagent (Beyotime #C2015) was added to the culture medium of monolayer cultured keratinocytes, and incubated at 37℃ for 30 min. Samples were imaged with confocal microscopy (Leica SP8).

**TUNEL assays**

TUNEL assays were carried out to assess the toxicity effects of Listerine® on oral epithelial tissues. Oral epithelial tissues were rinsed with ice-cold PBS, fixed in 4% (w/v) paraformaldehyde for 20 min at room temperature (RT), and rinsed with ice-cold PBS again. Then, samples were permeabilized with proteinase K for 30 min at 37°C and blocked with 3% H₂O₂ for 10 min. Samples were rinsed with PBS, and incubated in equilibration buffer for 10 min at RT. Then, samples were incubated with TUNEL reagents for 1 h at 37°C. Afterwards, samples were stained with DAPI and imaged with confocal microscopy (Leica SP8).

**H & E, immunohistochemistry (IHC), multi-label fluorescence staining, and Tunel assays**

Epithelial tissues or organoids were fixed in 4% (w/v) paraformaldehyde overnight at 4°C, dehydrated, and embedded in paraffin. 5-μm-thick histology sections were subject to H&E, IHC as well as multi-label fluorescence staining according to the manufacturer’s protocols. For 2D cultured keratinocyte staining, cells were cultured on glass slides and fixed in 4% (w/v) paraformaldehyde overnight at 4°C. Glass slides were then dehydrated and subject to H&E as well as IHC staining according to the manufacturer’s protocols. The primary antibodies adopted for IHC staining on epithelial tissues and organoids are listed in Supplementary Table 3. The tissue sections were subjected to TUNEL assays following the manufacturer's protocols (Servicebio #G1504).

**Biological transmission electron microscopy (TEM) assays**

Epithelial tissues, organoids and 2D cultured keratinocytes were collected fixed with glutaraldehyde solution (2.5%; Solarbio, Beijing, China) at 4℃ overnight. Samples were then sliced into thin chips and observed with transmission electron microscope (Hitachi, Tokyo, Japan).

**Quantitation and statistical analysis**

The data were presented as the mean ± standard deviation (SD). Each experiment was repeated in three duplicates. Statistically significant divergence between the means of two comparatives were assessed using a Student’s t test, while trails consisting of more than two comparatives were analyzed with a one-way analysis of variance followed by a Bonferroni’s multiple comparison test. Error bars in all figures represent standard deviation. All statistical analyses were performed in the SPSS statistics software (IBM 27.0). Bar plots and line charts were sketched using GraphPad Prism Version 8.

*p < 0.05, **p < 0.01, ***p < 0.001, respectively.

**Supplementary Table 1. Composition of organoid culture medium (PROs)**


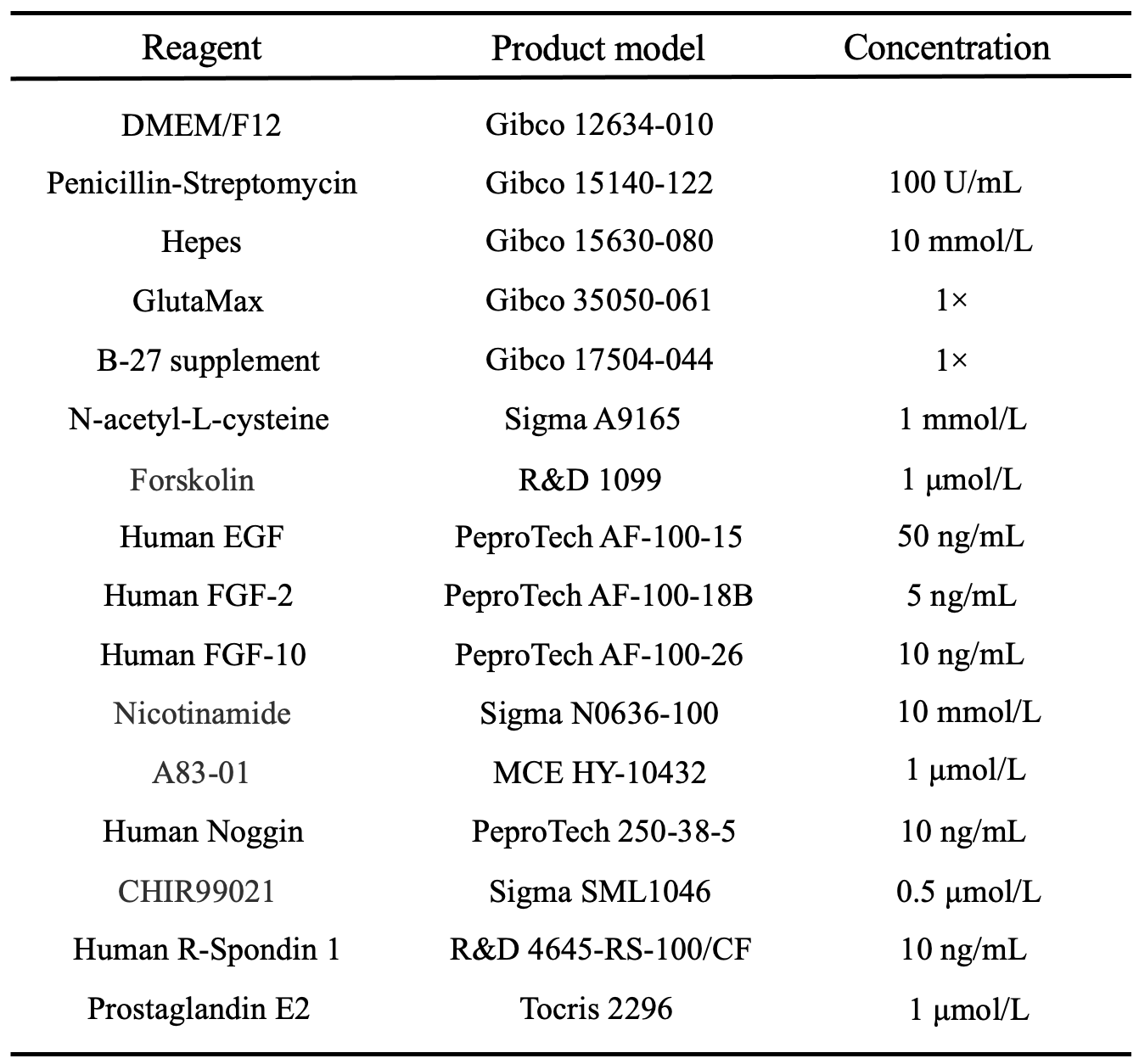


**Supplementary Table 2. Composition of organoid culture medium (EEOs)**


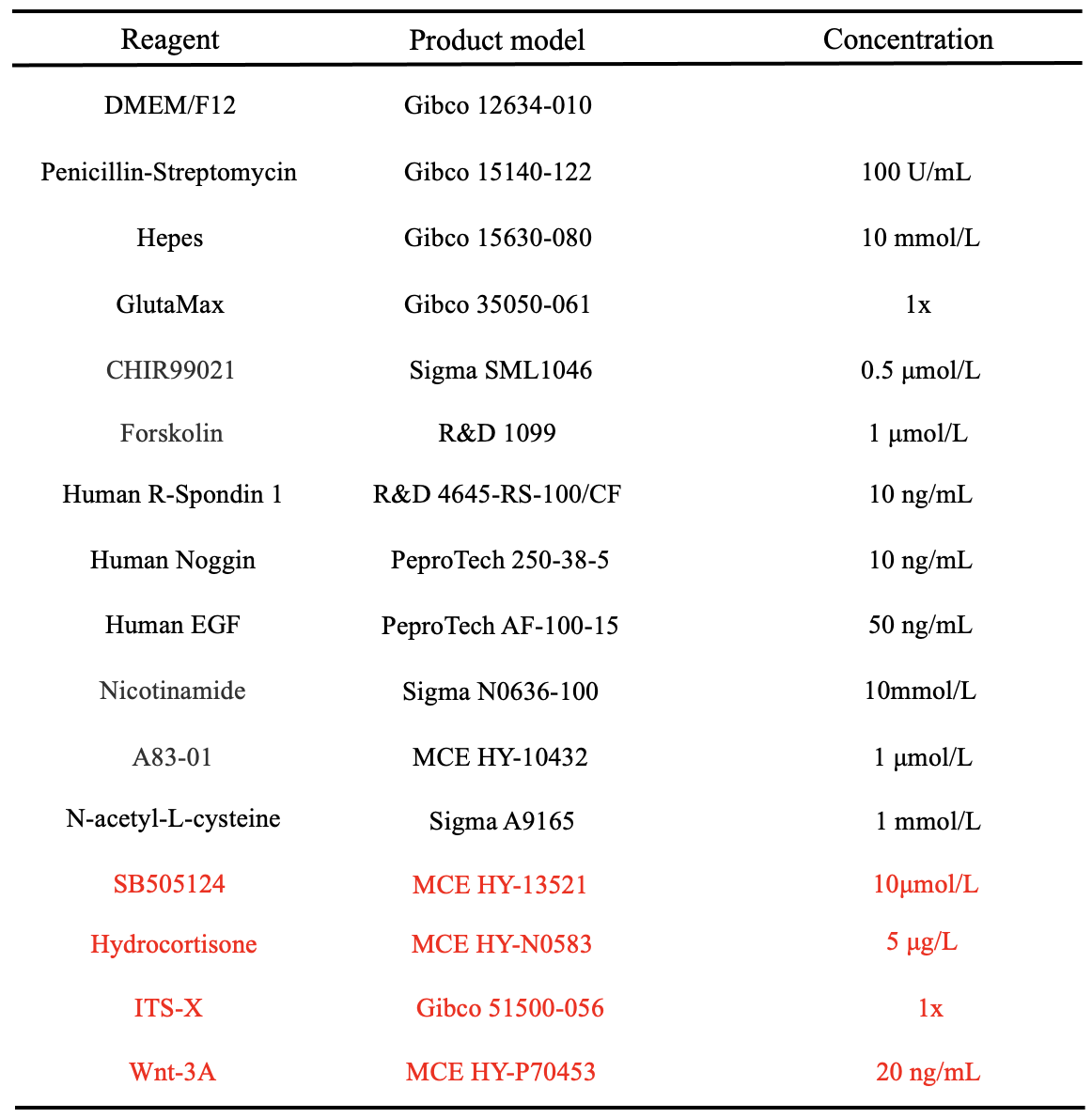


**Supplementary Table 3**

| species | Gene name | Forward primer (5'->3') | Reverse primer (5'->3') |
| --- | --- | --- | --- |
| Homo | VIM | GGACCAGCTAACCAACGACA | AAGGTCAAGACGTGCCAGAG |
| Homo | CDH1 | GGGGTCTGTCATGGAAGGTG | GAAACTCTCTCGGTCCAGCC |
| Homo | BMP2 | AAGCCAAACACAAACAGCGG | ATCAGCCAGAGGAAAAGGGC |
| Homo | NOTCH1 | GAGGCGTGGCAGACTATGC | CTTGTACTCCGTCAGCGTGA |
| Homo | FN1 | TGCACAACCAATGAAGGGGT | GTCCATTCCCCACGACCATT |
| Homo | ACTA2 | AGGAATACGATGAAGCCGGG | TGCTAGAGACAGAGAGGAGCA |
| Homo | COL4A1 | ACTGTGATTGCCCTCTACGC | GCTGTGGGCTGTGGTATTCT |
| Homo | SNAI2 | CAACGCCTCCAAAAAGCCAA | ACTCACTCGCCCCAAAGATG |
| Homo | FGFR1 | ATTTCTGCCTTGGCCCTACC | CTAGCGCAGTCTTTGGGGAA |
| Homo | TGFBR1 | CAGCCATGTGGGTCCTTTCT | TCCAGCACAGCAGAGTTACC |
| Homo | TGFB1 | ACCTGCCACAGATCCCCTAT | CTCCCGGCAAAAGGTAGGAG |
| Homo | CTNNB1 | GCTGGGACCTTGCATAACCT | CCAAGCATTTTCACCAGGGC |
| Homo | SMAD1 | CCACCCGTTTCCTCACTCTC | CCGCCTGAACATCTCCTCTG |
| Homo | SMAD2 | CGCTGGCCTGATCTTCACAG | AGTCATCCAGAGGCGGAAGT |
| Homo | SMAD3 | ATGGGGAGGGAGGGAGTTTT | TTTTTCCCGCTGTCCTGTGT |
| Homo | SMAD4 | AAACCATCCAGCATCCACCA | AGCCACAGGAATGTTGGGAA |
| Homo | SMAD5 | CCAGCCCAACAACACTCCTT | TAGGCAGGAGGAGGCGTATC |
| Homo | SMAD6 | GCCTGGGTTTGGTGTATGGT | CAGAAATCCGCTGAAGCCCT |
| Homo | SMAD7 | AGTATTGCTCACCCAGTGCC | AACACACAGGATGGGAGCAG |
| Homo | SMAD9 | GCAGGGAGTGGCTTGTATCA | TAGCACAGGCACCAAAGTCC |
| Homo | LORICRIN | GCCGTCCAAATAGATCCCCC | GCAAACCTCGGGTAGCATCA |
| Homo | IVL | CTGCCTCAGCCTTACTGTG | GCTCCTGATGGGTATTGACTG |
| Homo | LCE1A | CAAGAGTGGCTGAGATGCCC | AGTGAGTTTGTGGCTTGGGA |
| Homo | FZD7 | CCGTGTCGTTCTCTGTGCGA | GCGCGGTAGGGTAGGCAG |
| Homo | LRP5 | GACTTGGACAACCCGAGGTC | TCAATGGTGAGGTCGTTGGC |
| Homo | LRP6 | TGCTCCTACGACTTTCCTGC | GATGATGTCGGGGCTCTGTT |
| Homo | WNT7A | GCCTGGACGAGTGTCAGTTT | GTGGTACTGGCCTTGCTTCT |
| Homo | DKK1 | ACGCTATCAAGAACCTGCCC | GGGTACGGCTGGTAGTTGTC |
| Homo | KRTDAP | CACGCCCTCTTTGAGTCTATCA | GTTGCGCTCCTCAGTCCTT |
| Homo | KRT14 | TCCTTCGCACCAAGAACTGA | TATTGATTGCCAGGAGGGGG |
| Homo | TP63 | CGAGTAGAGGGGAACAGCCA | ATCCCTCCAACACAACTGCT |
| Homo | CCND1 | GAGGCGGAGGAGAACAAACA | GGAGGGCGGATTGGAAATGA |
| Homo | PCNA | AGCCGAAACCAGCTAGACTT | GTCCTTGAGTGCCTCCAACA |
| Homo | KRT6B | GTAGAGCTGGGATGCCCTCA | ATTCGCATGTCTGAGTGCTG |
| Homo | MKI67 | TCACGACCTCAAACTGGCTC | GAAATCGTGGCTGAAGGTGC |
| Homo | PI3K | TTTTGCTGTTCGGTGCTTGG | CCAAAAGCAGGCCAAACCTC |
| Homo | AKT | GGACAAGGACGGGCACATTA | CGACCGCACATCATCTCGTA |
| Homo | TJP1 | AAAGGCGGATGGTGCTACAA | CGCCTTCTGTGTCTGTGTCT |
| Homo | PTK2 | TGGGTCGGGAACTAGCTGTA | CAGTTTGGAGGTGCTCTGGT |
| Homo | OCLN | TCGTGAAGGCAAGTTTGTCAG | GCCAACGCCAACAGAAACAC |
| Homo | NOTCH1 | GAGGCGTGGCAGACTATGC | CTTGTACTCCGTCAGCGTGA |
| Homo | JAG2 | GCGATGAGTGTGTCCCCTAC | GCTGCCACAGTAGTTCAGGT |
| Homo | MAPK1 | GGCTGTTCCCAAATGCTGAC | AACTTGAATGGTGCTTCGGC |

**Supplementary Table 4**

| Antibody | Cat No | Manufacturer |
| --- | --- | --- |
| KRT1 | 185628 | Abcam |
| KRT2E | 21725-1-AP | Proteintech |
| KRT4 | 16572-2-AP | Proteintech |
| KRT5 | 64081 | Abcam |
| KRT6B | ab154313 | Abcam |
| KRT10 | 76318 | Abcam |
| KRT13 | ab16112 | Abcam |
| CDH1 | 60335-1-AP | Proteintech |
| TP63 | ab124762 | Abcam |
| MKI67 | ab16667 | Abcam |
| TGFB1 | 21898-1-AP | Proteintech |
| TGFBR1 | bs-0638R | Bioss |
| SMAD1/5/9 | ab66737 | Abcam |
| SMAD2/3 | 12570-1-AP | Proteintech |
| SMAD4 | 51069-1-AP | Proteintech |
| SNAI2 | 12129-1-AP | Proteintech |
| FGFR1 | 60325-1-AP | Proteintech |
| BMP2 | 66383-1-AP | Proteintech |
| EGFR | ab52894 | Abcam |
| CTNNB1 | ab32572 | Abcam |
| LUM | ab168348 | Abcam |
| VIM | ab8069 | Abcam |
| IVL | ab68 | Abcam |
| CD109 | 60179-1 | Proteintech |
| PTK2 | 40794 | Abcam |
| TJP1 | 66452-1 | Proteintech |
| OCLN | 27260-1 | Proteintech |
| DSP | 68364-1 | Proteintech |
| DSG3 | 29942-1 | Proteintech |
| LORICRIN | 55439-1 | Proteintech |
| NOTCH1 | 20687 | Proteintech |
| JAG1 | 66890 | Proteintech |
| PI3K | 4249 | CST |
| AKT | 9272 | CST |
| FZD7 | 16974 | Proteintech |
| GAPDH | 10494-1-AP | Proteintech |
